# Integrated Chemical Profiling and Toxicological Assessment of Total Polyphenols from *Cydonia oblonga* Mill. Reveals Potential Safety Concerns Under High-Dose Exposure

**DOI:** 10.3390/foods15203737

**Published:** 2026-10-20

**Authors:** Alifeiye Aikebaier, Alhar Baishan, Kayisaier Abudurousuli, Dilihuma Dilimulati, Yipaerguli Paerhati, Kuerbannisha Maitikasimu, Yilixiati Wusiman, Nazhakaiti Yusufujiang, Xiaoxiao Qiu, Wenting Zhou

**Affiliations:** 1 Department of Pharmacology, School of Pharmacy, Xinjiang Medical University, Urumqi 830017, China; alifeiye@stu.xjmu.edu.cn (A.A.); alhar@stu.xjmu.edu.cn (A.B.); yipaerguli@stu.xjmu.edu.cn (Y.P.); yilixiati@stu.xjmu.edu.cn (Y.W.); nzkt0110@stu.xjmu.edu.cn (N.Y.); qiuxiaoxiao@stu.xjmu.edu.cn (X.Q.); 2 Xinjiang Key Laboratory of Natural Medicines Active Components and Drug Release Technology, Urumqi 830017, China; 3 Xinjiang Key Laboratory of Biopharmaceuticals and Medical Devices, Urumqi 830017, China; 4 Engineering Research Center of Xinjiang and Central Asian Medicine Resources, Ministry of Education, Urumqi 830017, China; 5 School of Brain Science and Brain Medicine, Zhejiang University School of Medicine, Hangzhou 310058, China; 12518114@zju.edu.cn; 6 Xinjiang Institute of Materia Medica, Urumqi 830011, China; seher@sina.cn

**Keywords:** *Cydonia oblonga*, functional food safety, polyphenols, toxicological assessment, metabolomics, network toxicology, metabolic markers

## Abstract

The increasing consumption of polyphenol-enriched functional foods and dietary supplements highlights the importance of systematic safety evaluation of these bioactive ingredients under high-dose exposure conditions. This study comprehensively evaluated the toxicological responses associated with total polyphenols from *Cydonia oblonga* Mill. (TPCOM) and explored their potential biological mechanisms. Ultra-high-performance liquid chromatography coupled with quadrupole-Orbitrap mass spectrometry identified 89 selected phenolic and related constituents. Acute and 28-day repeated-dose oral toxicity studies were conducted with reference to OECD Guideline 423 and in accordance with OECD Guideline 407, respectively. High-dose TPCOM exposure resulted in dose-associated biochemical and histopathological alterations, with the liver and kidneys identified as major target organs. Integrated systems toxicology approaches, including network toxicology, molecular docking, molecular dynamics simulations, and untargeted plasma metabolomics, revealed potential molecular targets, metabolic disturbances, and candidate biomarkers associated with TPCOM exposure. These findings suggest that excessive intake of TPCOM may be associated with metabolic imbalance, stress-related signaling alterations, and organ injury responses. Further experimental validation is required to confirm the specific molecular pathways involved. This study provides a systematic framework for evaluating the safety of polyphenol-rich functional food ingredients under high-dose exposure conditions.

## 1. Introduction

*Cydonia oblonga* Mill., a medicinal and edible plant belonging to the Rosaceae family, has a long history of traditional pharmacological and culinary use worldwide [1,2]. Modern phytochemical and pharmacological studies indicate that its fruits are rich in polyphenolic compounds, such as catechins, quercetin derivatives, and chlorogenic acid. These components are considered the primary material basis responsible for its antioxidant, anti-inflammatory, hypoglycemic, and cardiovascular protective activities [3,4]. Consequently, total polyphenols from *Cydonia oblonga* Mill. (TPCOM) have demonstrated tremendous application potential in the development of functional foods, dietary supplements, and natural medicines. However, with the surge in the consumption of polyphenol-rich health products, their safety has increasingly become a focal point for academia and regulatory agencies [5,6]. Available toxicological evidence for *Cydonia oblonga* remains limited and appears to depend on the plant part, extraction procedure, and exposure conditions. Previous animal studies have reported relatively good tolerance of several *C. oblonga* preparations. An aqueous–methanolic seed extract was reported to be safe in mice at doses up to 4 g/kg, with no major alterations in blood chemistry during sub-chronic exposure [7], while a hydroalcoholic fruit extract showed no apparent acute oral toxicity in rats at doses up to 3000 mg/kg [8]. However, these findings cannot be directly extrapolated to polyphenol-enriched preparations because differences in plant material, extraction procedures, phytochemical composition, and administered dose may substantially influence toxicological outcomes.

Traditionally, natural polyphenols have often been labeled as entirely safe. However, emerging toxicological evidence indicates that polyphenols may exhibit hormesis in vivo dose–response [9,10,11]. They exert antioxidant and cytoprotective effects at physiological doses but may convert into pro-oxidants under high-dose or long-term, high-frequency exposure. This conversion can trigger a cascade accumulation of intracellular reactive oxygen species (ROS), leading to mitochondrial dysfunction, apoptosis, and severe damage to parenchymal organs, particularly the liver and kidneys [12,13,14]. Furthermore, high concentrations of polyphenol fractions may exert strong competitive inhibition or induction on phase I and II metabolic enzymes (the CYP450 family) in hepatocytes, causing a breakdown of metabolic homeostasis [15,16,17]. Despite the significant pharmacological value of TPCOM, there remains a substantial knowledge gap regarding its systematic toxicological risk assessment under acute and subacute exposure conditions, especially concerning target organ toxicity and the underlying molecular mechanisms. This limitation severely restricts the clinical translation and safe application of TPCOM.

Traditional toxicological evaluation systems, such as the OECD 423 acute toxicity test and the OECD 407 subacute toxicity test, are the gold standards for determining the median lethal dose (LD50), the no-observed-adverse-effect level (NOAEL), and macroscopic target organ toxicity of xenobiotics [18,19,20]. However, relying solely on phenotypic toxicological observations makes it difficult to comprehensively elucidate the multi-component and multi-target toxicological networks of complex plant extracts. Recently, the emergence of systems toxicology has provided a novel perspective to address this challenge. Network toxicology, combined with molecular docking and molecular dynamics simulations, can be used to predict plausible ligand–target interactions and to evaluate the dynamic stability of selected complexes at the molecular level [21,22,23]. Concurrently, untargeted metabolomics can sensitively capture early metabolic disorders and toxic biomarkers induced by chemical exposure from the macroscopic dimension of endogenous metabolite reprogramming [24,25]. The deep integration of multi-omics technologies provides a powerful tool for the comprehensive and multidimensional interpretation of the toxicological mechanisms of natural products [26].

Based on the aforementioned background, the present study proposes the hypothesis that excessive exposure to TPCOM may induce adverse biological responses distinct from its beneficial effects observed at nutritional levels. These responses may involve alterations in xenobiotic metabolism, oxidative stress-related processes, mitochondrial energy metabolism, and systemic metabolic homeostasis. To systematically evaluate the safety profile of TPCOM, this study first comprehensively characterized the chemical composition of TPCOM using UHPLC-QE-MS technology. Subsequently, acute and 28-day repeated-dose oral toxicity studies were conducted in a mouse model according to OECD guidelines to identify potential target organs and dose-associated toxicological responses. Finally, by integrating network toxicology, computational chemistry approaches (molecular docking and molecular dynamics simulations), and untargeted metabolomics strategies, this study explored potential molecular targets and metabolic alterations associated with high-dose TPCOM exposure. Rather than establishing definitive causal mechanisms, this integrated approach provides mechanistic insights into the biological responses associated with TPCOM-induced toxicity and contributes to a comprehensive understanding of the safety profile of polyphenol-rich functional food ingredients. Ultimately, this research aims to provide scientific evidence supporting the safety evaluation and rational application of TPCOM in the fields of functional foods and natural products.

## 2. Materials and Methods

### 2.1. Chemicals and Reagents

Fresh fruits of *Cydonia oblonga* Mill. were purchased from Yecheng County, Kashgar Prefecture, Xinjiang Uygur Autonomous Region, China. The plant material was authenticated as *Cydonia oblonga* Mill., belonging to the genus *Cydonia* Mill. of the family Rosaceae, by Professor Palida Abulizi from the Department of Natural Medicinal Chemistry and Pharmacognosy, College of Pharmacy, Xinjiang Medical University, and voucher specimens were deposited in our laboratory. Ethanol (analytical grade) and AB-8 macroporous adsorption resin utilized for plant extraction and purification were purchased from Sinopharm Chemical Reagent Co., Ltd. (Shanghai, China). Ultrapure water used in the experiments was freshly prepared utilizing a Milli-Q Advantage A10 ultrapure water system (Merck KGaA, Millipore, Darmstadt, Germany). Acetonitrile (ACN, LC-MS grade, CNW Technologies GmbH, Düsseldorf, Germany), isopropanol (IPA, LC-MS grade, CNW Technologies GmbH, Düsseldorf, Germany), and acetic acid (LC-MS grade, Sigma-Aldrich, St. Louis, MO, USA) utilized for UHPLC-QE-MS analysis were all of mass spectrometry grade. The Phenomenex Kinetex C18 chromatographic column (2.1 mm × 100 mm, 2.6 μm) employed for chromatographic separation was obtained from Phenomenex (Torrance, CA, USA). Reagents and consumables related to histopathological evaluation, including 10% neutral buffered formalin (NBF), routine hematoxylin and eosin (H&E) stains, and paraffin embedding materials, were all purchased from Thermo Fisher Scientific (China) Co., Ltd. (Shanghai, China). EDTA, sodium citrate, and heparinized anticoagulant tubes utilized for blood collection in in vivo experiments were acquired from Shandong Weigao Group Medical Polymer Co., Ltd. (Weihai, China). The detection of serum biochemical parameters (including ALT, AST, ALP, BUN, and CRE) was performed using a fully automated biochemical analyzer (BS-240VET) and matching commercial assay kits (Shenzhen Mindray Bio-Medical Electronics Co., Ltd., Shenzhen, China), with all biochemical measurements conducted strictly according to the manufacturer’s instructions.

### 2.2. Preparation of the Fruit Extract and LC-MS/MS Chemical Profiling

To prepare the total polyphenols from *Cydonia oblonga* Mill. (TPCOM), the dried fruits were ground into a fine powder and extracted with 60% (*v*/*v*) ethanol at a solid-to-liquid ratio of 1:40 (g/mL) at room temperature for 24 h under continuous stirring. The extraction procedure was repeated three times. The combined extracts were filtered through Whatman No. 1 filter paper and concentrated under reduced pressure at 40 °C using a rotary evaporator until complete removal of the ethanol. The resulting crude extract was subsequently purified using an AB-8 macroporous adsorption resin column. After sample loading, the column was sequentially eluted with purified water and 60% ethanol. The 60% ethanol fraction was collected, concentrated under reduced pressure, and lyophilized to obtain the TPCOM powder. The yield of the crude extract was 25.54%, and the final yield of the AB-8-purified fraction was 4.26%. The total phenolic content of the purified extract was determined using the Folin–Ciocalteu method and was 381.712 ± 0.529 mg gallic acid equivalents (GAE)/g dry weight.

The chemical composition of TPCOM was characterized using a Vanquish ultra-high-performance liquid chromatography system coupled with an Orbitrap Exploris 120 mass spectrometer (Thermo Fisher Scientific). Chromatographic separation was performed on a Phenomenex Kinetex C18 column (2.1 mm × 100 mm, 2.6 μm). The injection volume was 2 μL, and the autosampler temperature was maintained at 4 °C. The mobile phase consisted of 0.01% aqueous acetic acid (A) and an isopropanol-acetonitrile mixture (1:1, *v*/*v*) (B). Mass spectrometry data were acquired using Xcalibur software (version 4.4, Thermo Fisher Scientific) in data-dependent acquisition (DDA) mode. The electrospray ionization (ESI) source parameters were set as follows: spray voltages of 3.8 kV in positive ion mode and −3.4 kV in negative ion mode; capillary temperature of 320 °C; sheath gas flow rate of 50 arb; auxiliary gas flow rate of 15 arb; full MS resolution of 60,000 and MS/MS resolution of 15,000; and stepped normalized collision energies (SNCEs) of 20, 30, and 40.

The acquired raw MS data were processed using Compound Discoverer 3.1 software for peak detection, alignment, and feature extraction. Compound identification was performed based on accurate precursor ion mass, isotope distribution patterns, MS/MS fragmentation information, chromatographic retention time (RT), and spectral matching. For compounds assigned as Level 1, the acquired MS1 and MS/MS spectra and RT were matched with corresponding authentic reference standards in a reference spectral library. Only compounds showing concordant matching of MS1, MS/MS, and RT with the corresponding authentic standards were classified as Level 1. Database resources, including mzCloud, mzVault, and MassList, were additionally used to support spectral annotation and chemical characterization. The identified chemical constituents were subsequently classified according to their structural characteristics, and selected phenolic and related constituents were retained for subsequent toxicological analysis.

### 2.3. Experimental Animals and Ethical Statement

Specific pathogen-free (SPF) C57BL/6 mice were purchased from the Animal Experimental Center of Xinjiang Medical University. Mice used in the acute toxicity study were 6–8 weeks old and weighed 25 ± 2 g, whereas mice used in the 28-day repeated-dose toxicity study were 6–8 weeks old and weighed 18–22 g. Male mice aged 6–8 weeks and weighing 18–22 g were used for the untargeted metabolomics analysis.

All animals were acclimated for 7 days under standardized laboratory conditions at 23 ± 2 °C and 50% ± 10% relative humidity with a 12-h light/dark cycle and access to a standard laboratory diet and sterile water. Animals were allocated to the experimental groups using a random number table.

All animal experimental procedures were conducted in accordance with the Guide for the Care and Use of Laboratory Animals and with reference to the relevant OECD Guidelines for the Testing of Chemicals (TG 423 and TG 407). The experimental procedures were reviewed and approved by the Animal Ethics Committee of the First Affiliated Hospital of Xinjiang Medical University (Ethical Approval No. IACUC-20190226-22; approved on 26 February 2019).

Animals were randomly allocated to the experimental groups using a random number table. To minimize potential confounding, the order of treatment administration and outcome assessment was randomized across experimental groups. Personnel responsible for treatment administration were aware of group allocation, whereas biological samples were coded and investigators performing outcome assessment and data analysis were blinded to treatment allocation until completion of the analyses.

### 2.4. Acute Toxicity Test

The acute oral toxicity study was conducted with reference to OECD Guideline 423. Following acclimatization, animals were allocated using a random number table and fasted before dosing. TPCOM was prepared in distilled water and administered once by oral gavage at an administration volume of 20 mL/kg.

An initial high-dose test was conducted at 5000 mg/kg using six C57BL/6 mice, consisting of three males and three females. Four of the six animals died during the observation period, corresponding to a mortality rate of 66.7%. Because substantial mortality was observed at 5000 mg/kg, the dose was subsequently reduced to 2500 mg/kg. Six mice (three males and three females) were administered 2500 mg/kg TPCOM, and two of the six animals died, corresponding to a mortality rate of 33.3%. Because mortality persisted at 2500 mg/kg, the dose was further reduced to 1000 mg/kg for subsequent acute toxicity characterization.

At 1000 mg/kg, six mice (three males and three females) received a single oral administration of TPCOM. An additional six mice (three males and three females) served as the vehicle control group and received an equivalent volume of distilled water. No mortality occurred in the 1000 mg/kg treatment group during the 14-day observation period.

Following administration, animals were closely monitored during the initial post-dose period and were subsequently observed daily throughout the 14-day observation period. Clinical observations included mortality, general activity and behavior, fur condition, respiration, food intake, and other visible signs of toxicity. Body weight and food intake were also monitored during the observation period.

Humane endpoints were predefined to minimize unnecessary animal suffering. Animals exhibiting severe reduction in activity, inability to eat or drink, or persistent respiratory distress were to be removed from the study and humanely euthanized.

At 5000 mg/kg, clinical signs observed before death included reduced activity, restlessness/agitation, and piloerection, and abnormal changes in body weight were observed during the observation period. Gross necropsy of animals that died at this dose revealed marked dark discoloration of the liver. At the end of the 14-day observation period, surviving animals underwent blood collection followed by necropsy and histopathological examination.

### 2.5. Subacute Toxicity Test

Based on the results of the acute toxicity study, a 28-day repeated-dose oral toxicity study was conducted in accordance with OECD Guideline 407. Forty-eight C57BL/6 mice were randomly assigned to a vehicle control group or one of three TPCOM treatment groups (125, 250, or 500 mg/kg/day), with 12 mice per group (six males and six females). The control group received an equal volume of distilled water, whereas the treatment groups received TPCOM at the corresponding doses by oral gavage once daily for 28 consecutive days. All mice were fasted for 2 h before daily administration, with free access to water.

Throughout the study, clinical signs, including changes in locomotor activity, fur condition, respiration, neurological status, and gastrointestinal responses, as well as mortality, were monitored twice daily. Body weight and food intake were recorded regularly during the experimental period. On day 29, following an 18-h fasting period, blood samples were collected via the retro-orbital venous plexus. The mice were subsequently euthanized, and a complete necropsy was performed for further analyses.

### 2.6. Hematological, Biochemical, and Histopathological Evaluation

Hematological, coagulation, and biochemical analyses: Blood samples were divided according to the requirements of the different analyses. EDTA-anticoagulated whole blood was used for hematological measurements. Sodium citrate-anticoagulated blood was centrifuged to obtain plasma for coagulation analysis, including prothrombin time (PT) and activated partial thromboplastin time (APTT). Separate blood aliquots were processed to obtain serum for biochemical measurements. A fully automated hematology analyzer (KX-21, Sysmex, Japan) was used to measure hematological parameters, including WBC, Neu, Lym, Mon, Eos, Bas, RBC, HGB, HCT, MCV, MCH, MCHC, RTC, PLT, MPV, and PDW. Coagulation parameters, including PT and APTT, were measured using plasma samples. Serum biochemical parameters, including TP, ALT, AST, ALB, A/G ratio, ALP, TCHO, TG, BUN, GLU, and CRE, were measured using an automated biochemical analyzer.

Organ indices and histopathology: During necropsy, macroscopic organ lesions were carefully observed. The heart, liver, spleen, kidneys, and lungs were isolated and weighed to obtain their absolute weights, which were used to calculate the relative organ weights (organ index = organ weight/fasted body weight × 100%). Subsequently, the tissue samples were fixed in 10% neutral buffered formalin (NBF), routinely embedded in paraffin, sectioned (5 μm), and stained with hematoxylin and eosin (H&E). No standardized semiquantitative lesion-scoring system was applied in the present study. The sections were evaluated for cell necrosis, inflammation, and other potential toxicological damage under a microscope using a panoramic slide scanner (Wuhan Servicebio Technology Co., Ltd., Wuhan, Hubei, China).

### 2.7. Network Toxicology Analysis

#### 2.7.1. Screening of Potential Active Compounds and Toxic Components

Based on the chemical constituents identified by UHPLC-QE-MS, a rigorous three-step screening strategy was employed to select phenolic and related constituents for subsequent toxicological analysis. First, pharmacokinetic properties were evaluated using the TCMSP and SwissADME databases. Compounds with oral bioavailability (OB) ≥ 30% and drug-likeness (DL) ≥ 0.18 were retained; for compounds lacking complete OB/DL data, those with “High” gastrointestinal absorption and satisfying at least two of Lipinski’s Rule of Five criteria were included. Second, the toxicity potential of the screened compounds was predicted using ADMETlab 3.0, with particular focus on hepatotoxicity and nephrotoxicity. Third, the InChIKey of each compound was used as a query to cross-validate hepatotoxic and nephrotoxic associations in the Comparative Toxicogenomics Database (CTD) and PubChem. Through this multi-dimensional screening process, 89 selected phenolic and related constituents (numbered P1–P89) were ultimately selected as the principal material basis for network toxicology analysis (Supplementary Figure S1 and Supplementary Table S2). The complete screening workflow and criteria are detailed in Supplementary Table S5.

#### 2.7.2. Target Prediction of Toxic Components and Acquisition of Hepato-Renal Injury Targets

To determine the molecular targets of the potential toxic components in TPCOM, the screened hepato-renal toxicity compounds were submitted to the SEA, SwissTargetPrediction, TargetNet, and ChEMBL databases for target prediction. After merging, converting standard gene names, and removing duplicates from the prediction results of each database, a potential target set for TPCOM toxic components was constructed. Meanwhile, to obtain disease targets related to organ toxicity, comprehensive searches were conducted in the GeneCards, CTD, TTD, and OMIM databases using “drug-induced liver injury”, “hepatotoxicity”, “drug-induced kidney injury”, and “nephrotoxicity” as keywords. The search results from these four databases were integrated, and duplicate genes were removed to construct a hepato-renal toxicity disease target set. Finally, an online Venn diagram tool was used to intersect the TPCOM toxic component target set with the disease target set. The overlapping genes were defined as the core targets for TPCOM-induced potential hepato/nephrotoxicity, which were used for subsequent protein–protein interaction (PPI) network construction and enrichment analysis.

#### 2.7.3. Protein–Protein InteractionProtein-Protein Interaction (PPI) Network Construction and Core Target Screening

The screened overlapping targets for hepato/nephrotoxicity were imported into the STRING database (version 11.5) to construct a PPI network. In the search parameters, the species was strictly limited to “Homo sapiens”, and the minimum required interaction score threshold was set to >0.4 to ensure the reliability of the network, while disconnected nodes were hidden. Subsequently, the extracted PPI network data were imported into Cytoscape software (version 3.9.1) for visualization and topological parameter analysis. The built-in plugin CytoHubba (using algorithms including MCC, MNC, and Degree) was used to evaluate the topological importance of each node. Simultaneously, the MCODE plugin was applied to extract highly interconnected protein clustering modules within the PPI network. By integrating these topological features, the core hub targets mediating the potential hepato-renal toxicity of TPCOM were precisely identified. The human species setting was selected because the compound-target prediction and toxicity-associated databases used in the upstream network analysis predominantly rely on human gene/protein annotations, allowing consistent target integration across databases.

#### 2.7.4. GO Functional and KEGG Pathway Enrichment Analysis

The identified core targets were imported into the DAVID database for Gene Ontology (GO) functional annotation and Kyoto Encyclopedia of Genes and Genomes (KEGG) pathway enrichment to explore the potential biological mechanisms underlying hepato-renal toxicity.analyses The GO analysis covered three dimensions: biological process (BP), cellular component (CC), and molecular function (MF). The parameters for enrichment analysis were strictly set as: *p*-value < 0.05, minimum gene overlap ≥ 3, and fold enrichment ≥ 1.5. Additionally, the Benjamini–HochbergBenjamini-Hochberg method was adopted for multiple testing correction (Q-value < 0.05) to effectively eliminate false-positive results. The significantly enriched results were visualized using bar charts and bubble charts generated by the online bioinformatics platform SRplot. For highly significant core pathways, target mapping and coloring analysis were performed using the official tools of the KEGG database. Finally, a core pathway network map related to hepato-renal toxicity was constructed.

#### 2.7.5. Molecular Docking to Evaluate Predicted Ligand–Target Interactions

To evaluate the potential interactions between toxicologically relevant TPCOM components and the core targets, molecular docking simulations were performed using AutoDock Vina software (version 1.2.7) [27,28].

For the hepatotoxicity-related analysis, 12 candidate compounds were initially identified through network toxicology. To prioritize representative compounds for subsequent molecular docking, the connectivity of each compound node in the compound–target network was evaluated using its Degree value, defined as the number of candidate targets connected to that compound. The six compounds with the highest Degree values (compounds 66, 16, 71, 43, 15, and 41), which were connected to 660, 648, 643, 633, 608, and 608 candidate targets, respectively, were selected as representative hepatotoxicity-related compounds for docking against the six hub targets. This network-based prioritization was intended to select highly connected representative compounds for computational evaluation and did not exclude the potential toxicological relevance of the remaining candidate compounds.

Before docking, the three-dimensional structures of the receptor proteins were prepared by removing crystallographic water molecules and original ligands, adding polar hydrogen atoms, and assigning charges. The prepared receptor and ligand structures were subsequently converted into PDBQT format. The docking search space was defined individually for each target using target-specific grid centers and box dimensions. The receptor structure identifiers were AKT1 (1UNQ), CYP2E1 (3E6I), SRC (6C4S), NDUFS7 (O75251), NDUFS3 (O75489), NDUFV2 (P19404), JUN (1JNM), MAPK3 (4QTB), CYP3A4 (5VCC), RELA (7O6M), and MAPK1 (8AOJ). AutoDock Vina was run with an exhaustiveness value of 8. Detailed grid-center coordinates and box dimensions for each receptor are provided in Supplementary Table S6. The AutoDock Vina docking score (kcal/mol) was used to compare the predicted favorability of ligand–target interactions. More negative docking scores were considered to indicate more favorable predicted interactions. Based on the criteria adopted in this study, docking scores of ≤−4.25 kcal/mol, ≤−5.00 kcal/mol, and ≤−7.00 kcal/mol were considered indicative of potential binding activity, favorable predicted binding, and particularly strong predicted interactions, respectively [29,30,31]. Finally, PyMOL (version 3.1.6.1) and Discovery Studio 2019 were used to visualize representative docking poses and predicted amino acid residue interactions.

#### 2.7.6. Molecular Dynamics Simulations to Evaluate Binding Stability

To further evaluate the dynamic stability of representative ligand–receptor interactions identified through the integrated molecular docking and network toxicology analyses, eight representative ligand–target complexes were selected for 100-ns molecular dynamics (MD) simulations. The selection of these complexes was based on an integrated consideration of the molecular docking results, the network-topological importance of the corresponding targets, and the toxicological relevance of the ligand–target pairs, rather than solely on the absolute docking score. Accordingly, the docking-energy threshold used to characterize strong predicted interactions was not applied as an exclusive criterion for MD selection.

MD simulations were performed using GROMACS 2022.3. The AMBER99SB-ILDN force field was employed for receptor proteins, while the topological parameters for the small-molecule ligands were generated using Sobtop in combination with the GAFF force field. Each ligand–receptor complex was placed in a periodic cubic simulation box with a minimum distance of 1.0 nm between the protein and the box boundary and was solvated using the TIP3P water model. Na^+^ and Cl^−^ ions were subsequently added to neutralize the system.

Prior to the production simulation, energy minimization was performed using the steepest-descent algorithm to eliminate unfavorable steric contacts. Position restraints were then applied to the ligand–receptor complexes, followed by sequential equilibration under the NVT ensemble at 300 K for 1000 ps and the NPT ensemble at 1 bar and 300 K for 1000 ps. After equilibration, the position restraints were removed, and an unrestrained 100-ns production simulation was performed under the NPT ensemble. The cutoff distance for non-bonded interactions was set to 1.2 nm, and all bonds involving hydrogen atoms were constrained using the LINCS algorithm.

Following completion of the simulations, periodic boundary conditions were corrected using the gmx trjconv module. The dynamic behavior and structural stability of the complexes were evaluated using root-mean-square deviation (RMSD), root-mean-square fluctuation (RMSF), solvent-accessible surface area (SASA), radius of gyration (Rg), and intermolecular hydrogen-bond analyses. Free-energy landscapes were further constructed to characterize conformational sampling. In addition, molecular mechanics/Poisson–Boltzmann surface area (MM/PBSA) calculations were performed to estimate the binding free energy of the ligand–receptor complexes and to assess the contributions of different energetic components and key amino acid residues.

### 2.8. Untargeted Metabolomics Analysis Based on UHPLC-QE-MS

Male C57BL/6 mice were randomly divided into a control group (*n* = 3) and an experimental group (*n* = 3). The control group received an equal volume of distilled water via oral gavage, whereas the experimental group received a single oral dose of 500 mg/kg TPCOM. At 60 min post-administration, 150 µL of blood was collected from the retro-orbital venous plexus into heparinized anticoagulant tubes. The samples were centrifuged at 3500 rpm for 15 min to separate the plasma, which was then stored at −80 °C until further use. Subsequently, the plasma samples were subjected to untargeted metabolomics scanning analysis in both positive and negative ion modes using an ultra-high-performance liquid chromatography-high resolution mass spectrometry system (UHPLC-QExactive Focus; Thermo Fisher Scientific, Bremen, Germany).

The raw mass spectrometry data were imported into Compound Discoverer 3.1 (CD 3.1) software for peak extraction, peak alignment, and quantitative analysis. The quantitative results were normalized using the total peak area. The qualitative identification of metabolites was based on precise matching with the mzCloud, mzVault, and MassList databases. Quality-control (QC) samples were prepared by pooling equal volumes of all experimental plasma samples. Before analysis of the study samples, three consecutive QC injections were performed to monitor the initial instrument status and equilibrate the LC-MS/MS system. During the analytical sequence, QC samples were interspersed among the study samples to monitor system stability, and study samples were injected in a randomized order to minimize potential run-order and batch effects. Pairwise correlation analysis among QC samples was performed to evaluate analytical reproducibility. The QC samples showed high pairwise correlations, with R^2^ values ranging from 0.990 to 0.991 in negative-ion mode and from 0.990 to 0.993 in positive-ion mode. QC clustering in principal component analysis was also examined as an indicator of analytical stability. To ensure data quality, background noise and redundant data were systematically removed, and features with a coefficient of variation (CV) ≥ 30% in the QC samples were excluded from downstream analysis.

After evaluating the overall distribution trend of the samples using principal component analysis (PCA) on the normalized data, partial least squares-discriminant analysis (PLS-DA) was employed to assess the differences between groups. The screening criteria for candidate differential metabolites were set as variable importance in projection (VIP) > 1.0, nominal *p* < 0.05, and fold change (FC) > 1.5 or <0.667. To account for multiple testing, metabolite-level *p* values were additionally adjusted using the Benjamini–Hochberg false discovery rate (FDR) procedure. Metabolites with q < 0.05 were considered FDR-confirmed, whereas metabolites meeting the VIP, FC, and nominal *p*-value criteria were retained as exploratory candidate differential metabolites for subsequent hypothesis-generating analyses.

### 2.9. Statistical Analysis

All experimental data are expressed as the mean ± standard error of the mean (SEM). All statistical analyses and visual chart plotting were performed using GraphPad Prism software (version 10.6.1; GraphPad Software, LLC, San Diego, CA, USA). The normal distribution characteristics of the data were first evaluated using the Shapiro–WilkShapiro-Wilk test. For data conforming to a normal distribution, comparisons between two groups were performed using an independent samples *t*-test, while comparisons among multiple groups were conducted using a one-way analysis of variance (ANOVA). If the ANOVA results showed statistical significance, Tukey’s or Bonferroni’s post-hoc test was further applied for multiple comparisons between groups. For data not conforming to a normal distribution, non-parametric test methods were used (i.e., the Mann–WhitneyMann-Whitney U test for comparisons between two groups and the Kruskal–WallisKruskal-Wallis H test for comparisons among multiple groups). Longitudinal body-weight data were analyzed using two-way repeated-measures ANOVA, with time as the within-subject factor and treatment as the between-subject factor. The main effects of time and treatment, as well as the time × treatment interaction, were evaluated. A significance level of *p* < 0.05 was considered statistically significant. For endpoints measured in both male and female mice in the 28-day repeated-dose study, two-way ANOVA was additionally performed with sex and treatment as fixed factors to evaluate the main effects of sex and treatment and the sex × treatment interaction. A statistically significant interaction term was interpreted as evidence that the treatment response differed between male and female mice.

## 3. Results

### 3.1. Chemical Profile of the Cydonia oblonga Mill. Fruit Extract

**Figure 1:**
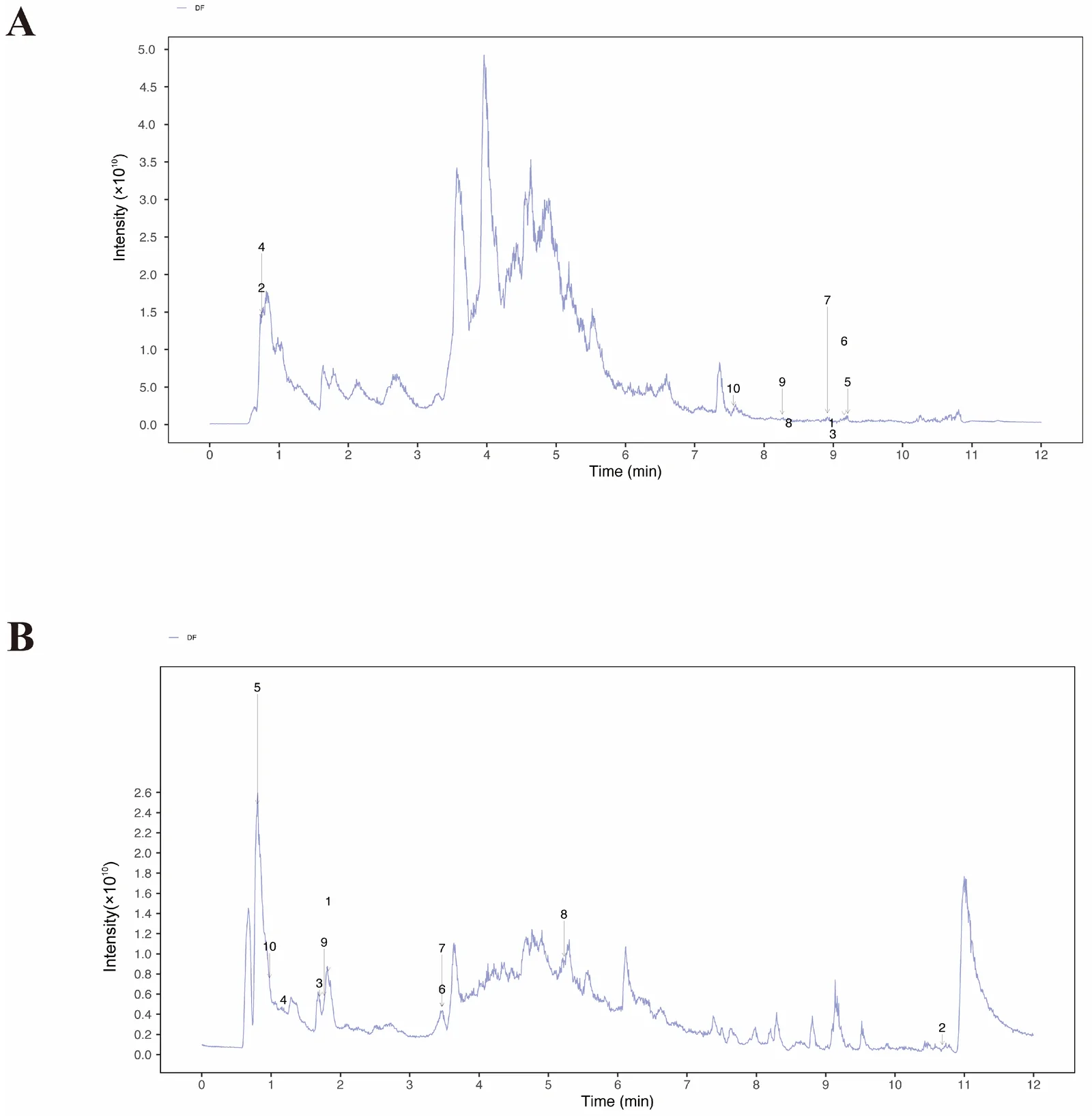
UHPLC-Q Exactive MS total ion current (TIC) chromatograms of *Cydonia oblonga* Mill. extract. **(A) Positive-ion mode (ESI+); (B) negative-ion mode (ESI−). Numbers indicate selected peaks, with numbering assigned independently for each ionization mode. The corresponding compound annotations are provided in Supplementary Table S7.**

The chemical profile of TPCOM was comprehensively characterized using ultra-high-performance liquid chromatography coupled with quadrupole-Orbitrap high-resolution mass spectrometry (UHPLC-QE-MS). The total ion current (TIC) chromatograms of the TPCOM extract acquired in positive- and negative-ion modes are shown in Figure 1. A total of 468 chemical constituents were identified based on accurate precursor ion masses, isotope distribution patterns, MS/MS fragmentation characteristics, chromatographic retention times, and spectral matching. All 468 compounds were classified as Level 1 identifications based on concordant matching of MS1, MS/MS, and RT with corresponding authentic reference standards. Among these identified constituents, 89 selected phenolic and related constituents were further selected as candidate compounds for subsequent toxicological analysis.

From the 468 identified constituents, 89 selected phenolic and related constituents (numbered P1–P89) were further selected using a rigorous three-step screening strategy (see Section 2.7.1 and Supplementary Figure S1). This strategy integrated pharmacokinetic properties (oral bioavailability and drug-likeness), toxicity potential prediction via ADMETlab 3.0, and literature-based toxicological associations from the Comparative Toxicogenomics Database (CTD) and PubChem. These 89 selected phenolic and related constituents are primarily phenolic acids, flavonoids, coumarins, and phenylpropanoids, and were considered the principal bioactive and potentially toxic components of TPCOM.

Relative quantitative profiling was performed based on normalized peak areas from the UHPLC-QE-MS data. A total of 89 selected phenolic and related constituents exhibited substantial variation in abundance. Notably, scopolin showed the highest relative MS response, accounting for approximately 73.5% of the summed normalized MS signal among the 89 selected compounds under the analytical conditions used. This value represents relative signal abundance and should not be interpreted as an absolute concentration or mass fraction of the TPCOM preparation. Other relatively abundant polyphenols included several phenolic acid derivatives, rutin, magnolioside, and quercetin. These relative abundance data were used to characterize the chemical profile of TPCOM and were not used as the sole criterion for selecting compounds for subsequent network toxicology or molecular docking analyses.

The complete identification information for all 468 Level 1 compounds, together with the detailed relative quantitative data, abundance ranking, and classification of the 89 selected phenolic and related constituents, is provided in Supplementary Tables S1 and S2, respectively. This systematic chemical characterization provides an analytical basis for the subsequent toxicological analyses of TPCOM. These constituents were selected using an integrated screening strategy incorporating pharmacokinetic properties, predicted toxicity, and toxicological evidence from CTD and PubChem.

### 3.2. Acute and Subacute Toxicity Evaluation of TPCOM

**Figure 2:**
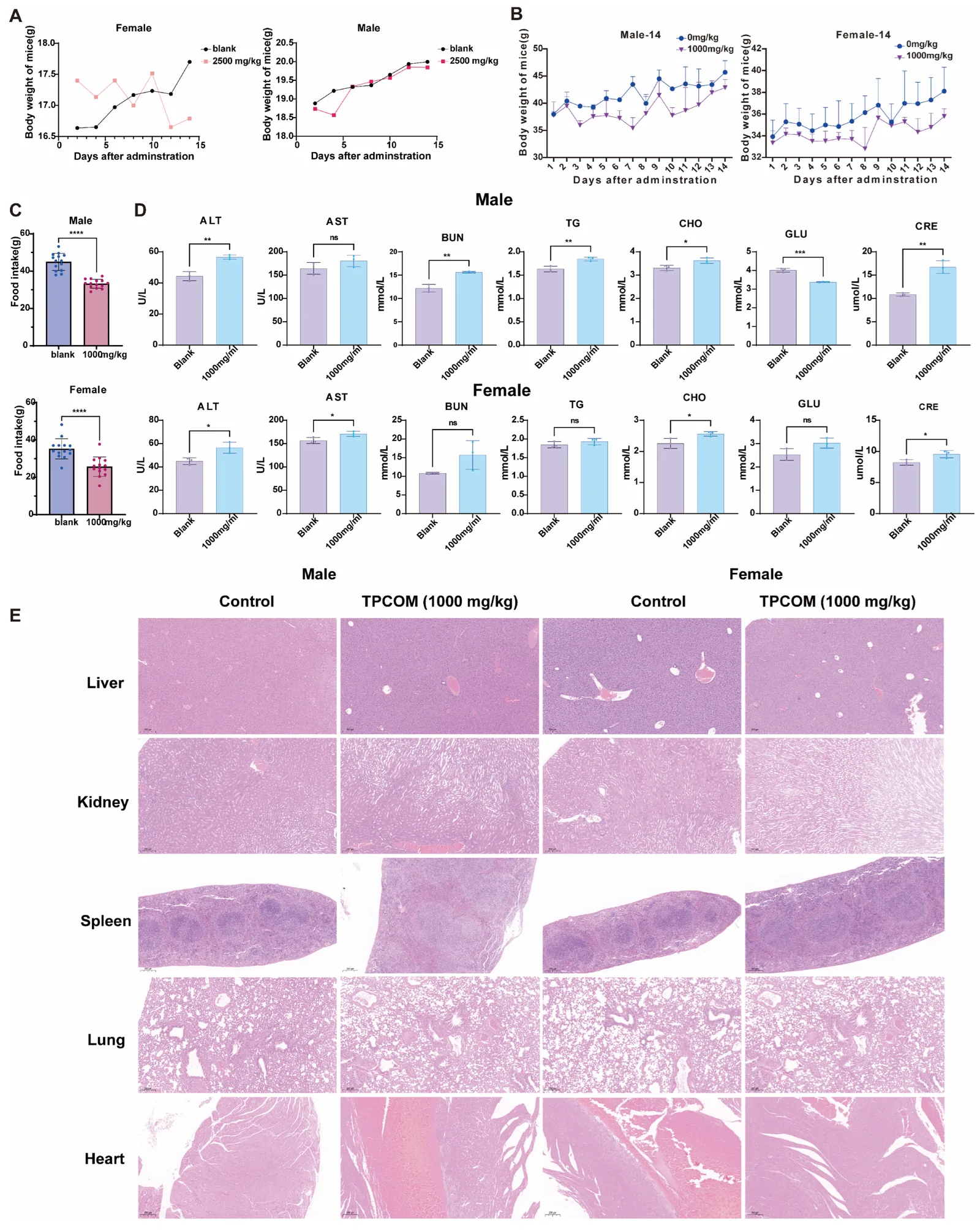
(**A**) Body weight changes of male and female mice during the acute toxicity dose-finding phase at 2500 mg/kg. (**B**) Dynamic body weight curves of mice over 14 days in the main acute test (1000 mg/kg). (**C**) Cumulative food intake of both sexes during the main acute test. (**D**) Serum biochemical parameters (ALT, AST, BUN, TG, CHO, GLU, and CRE) of male and female mice following acute exposure (1000 mg/kg). (**E**) Representative H&E-stained histopathological sections of core organs (liver, kidney, spleen, lung, and heart) from the control and TPCOM-treated (1000 mg/kg) groups. Data are presented as mean ± SEM. * *p* < 0.05, ** *p* < 0.01, *** *p* < 0.001, **** *p* < 0.0001; ns, not significant.

As illustrated in Figure 2, acute exposure to high doses of TPCOM resulted in evident toxic responses. In the initial high-dose test, a single oral administration of 5000 mg/kg TPCOM resulted in the death of four of six mice (66.7%) during the observation period. Clinical signs preceding death included reduced activity, restlessness/agitation, and piloerection, together with abnormal changes in body weight during the observation period. Gross necropsy of the animals that died at this dose revealed marked dark discoloration of the liver. Because of the substantial mortality observed at 5000 mg/kg, the dose was subsequently reduced to 2500 mg/kg. At 2500 mg/kg, two of six mice died, corresponding to a mortality rate of 33.3%. Body weight monitoring during the 2500 mg/kg dose-finding phase (Figure 2A) revealed a marked reduction in female mice accompanied by early mortality, whereas surviving male mice showed fluctuations in body weight. Because mortality persisted at 2500 mg/kg, the dose was further reduced to 1000 mg/kg for subsequent acute toxicity characterization.

At 1000 mg/kg, no mortality was observed during the 14-day observation period. Two-way repeated-measures ANOVA of longitudinal body-weight data showed a significant treatment effect in male mice [F(1,4) = 23.01, *p* = 0.0087], whereas the treatment × time interaction was not significant [F(13,52) = 0.42, *p* = 0.9573]. In female mice, a significant time effect was observed [F(13,52) = 7.77, *p* < 0.0001], while the treatment × time interaction was not significant [F(13,52) = 1.44, *p* = 0.1726] (Figure 2B). Cumulative food consumption was significantly reduced in the treatment group (*p* < 0.0001, Figure 2C). Serum biochemical analysis (Figure 2D) revealed alterations in hepatic- and renal-function-related parameters. ALT levels were significantly elevated in both male (*p* < 0.01) and female (*p* < 0.05) mice, while CRE levels were also significantly increased in both sexes (*p* < 0.01 and *p* < 0.05, respectively). Male mice additionally showed decreased GLU levels (*p* < 0.001), increased TG levels (*p* < 0.01), increased CHO levels (*p* < 0.05), and increased BUN levels (*p* < 0.01). Histopathological examination (Figure 2E) revealed disorganization of hepatic cords, cytoplasmic rarefaction, and mild hepatocellular degeneration, together with mild swelling of renal tubular epithelial cells. No severe parenchymal necrosis was observed in the spleen, lungs, or heart at this dose. Collectively, these findings indicate that acute high-dose TPCOM exposure was associated with biochemical and histopathological alterations, particularly involving the liver and kidneys, together with changes in glucose- and lipid-metabolism-related parameters.

**Figure 3:**
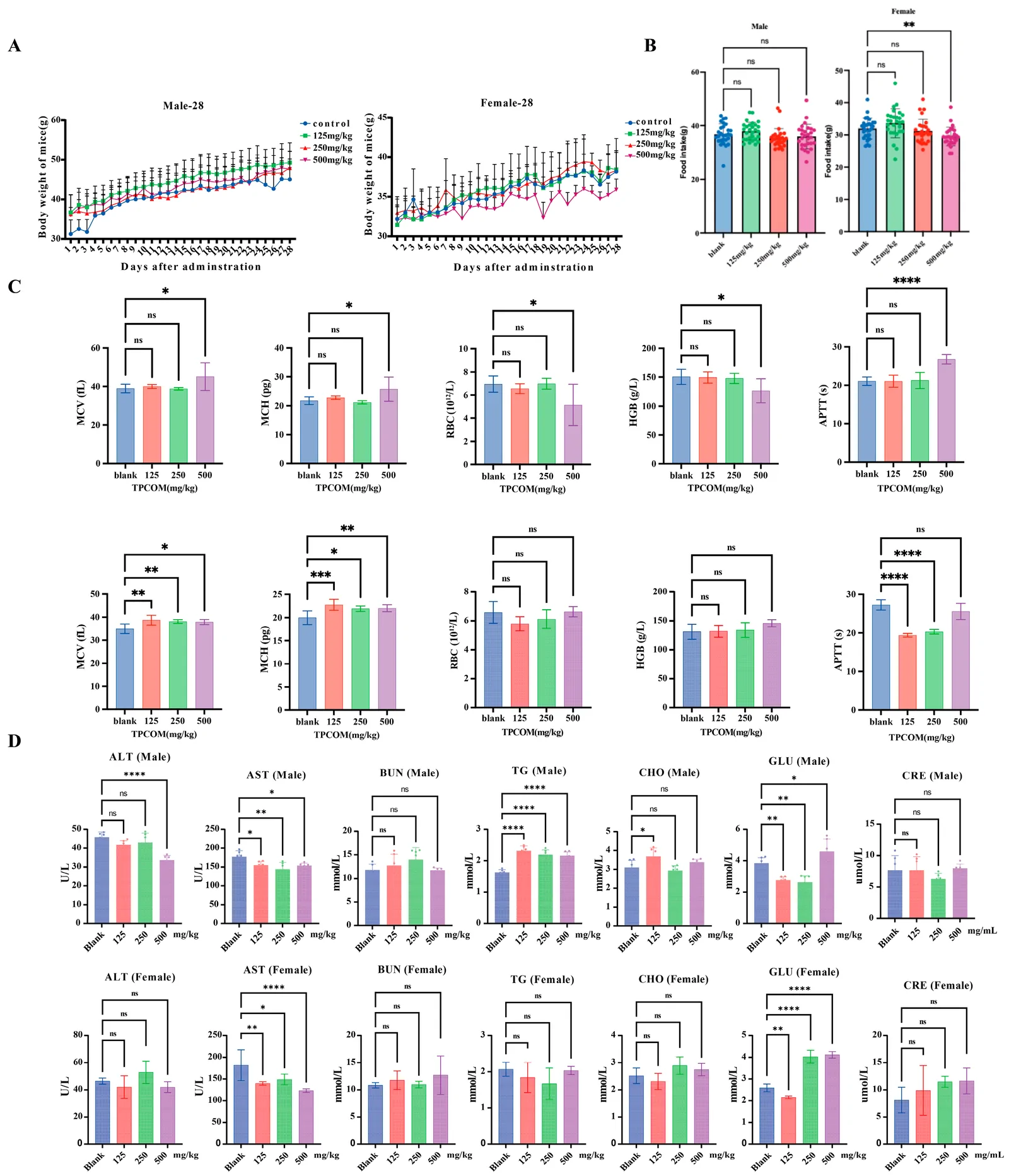
Subacute (28-day) toxicity evaluation of TPCOM in C57BL/6 mice. (**A**) Dynamic body weight curves of male and female mice during the 28-day repeated-dose administration across different groups. (**B**) Cumulative food intake of both sexes in each dose group. (**C**) Hematological parameters (including erythrocyte indices and coagulation function marker (APTT)) following subacute exposure. (**D**) Serum biochemical indicators reflecting hepato-renal function and glucolipid metabolism in male and female mice. * *p* < 0.05, ** *p* < 0.01, *** *p* < 0.001, **** *p* < 0.0001; ns, not significant.

The 28-day repeated-dose oral toxicity study (Figure 3) further characterized the toxicological responses associated with repeated TPCOM exposure. Regarding growth performance, repeated-measures analysis of the longitudinal body-weight data showed significant main effects of treatment and time in male mice (treatment: F(3,20) = 7.45, *p* = 0.0015; time: F(27,540) = 14.45, *p* < 0.0001), whereas the time × treatment interaction was not significant (F(81,540) = 0.23, *p* > 0.05). In female mice, significant main effects of treatment and time were also observed (treatment: F(3,20) = 34.76, *p* < 0.0001; time: F(27,540) = 12.09, *p* < 0.0001), with no significant time × treatment interaction (F(81,540) = 0.49, *p* > 0.05) (Figure 3A). Cumulative food consumption was significantly decreased in female mice treated with 500 mg/kg/day (*p* < 0.01, Figure 3B). Hematological analysis (Figure 3C) revealed increased mean corpuscular volume (MCV) and mean corpuscular hemoglobin (MCH) levels (*p* < 0.05 or *p* < 0.01), without significant changes in RBC or HGB levels. APTT showed distinct treatment-related response patterns between male and female mice. Two-way ANOVA demonstrated a significant sex × treatment interaction for APTT (F(3,40) = 20.79, *p* < 0.0001), indicating that the effect of TPCOM treatment on this coagulation parameter differed between sexes.

Serum biochemical analysis (Figure 3D) demonstrated alterations in glucose- and lipid-associated parameters after repeated TPCOM administration. Male mice showed significantly increased TG levels across all treatment groups (*p* < 0.0001), accompanied by decreased GLU levels (*p* < 0.01), whereas female mice exhibited significantly increased GLU levels at the medium and high doses (*p* < 0.0001). Two-way ANOVA further demonstrated significant sex × treatment interactions for GLU (F(3,40) = 27.70, *p* < 0.0001) and TG (F(3,40) = 9.74, *p* < 0.0001), indicating that the treatment-related responses of these metabolic parameters differed between male and female mice. Significant sex × treatment interactions were also detected for CHO, ALP, TP, AST/ALT, AST, and ALT (*p* < 0.05). Decreased ALT and AST levels were observed in several treatment groups of both sexes; however, these decreases were not considered direct evidence of hepatocellular injury in isolation, and their toxicological significance remains uncertain.

**Figure 4:**
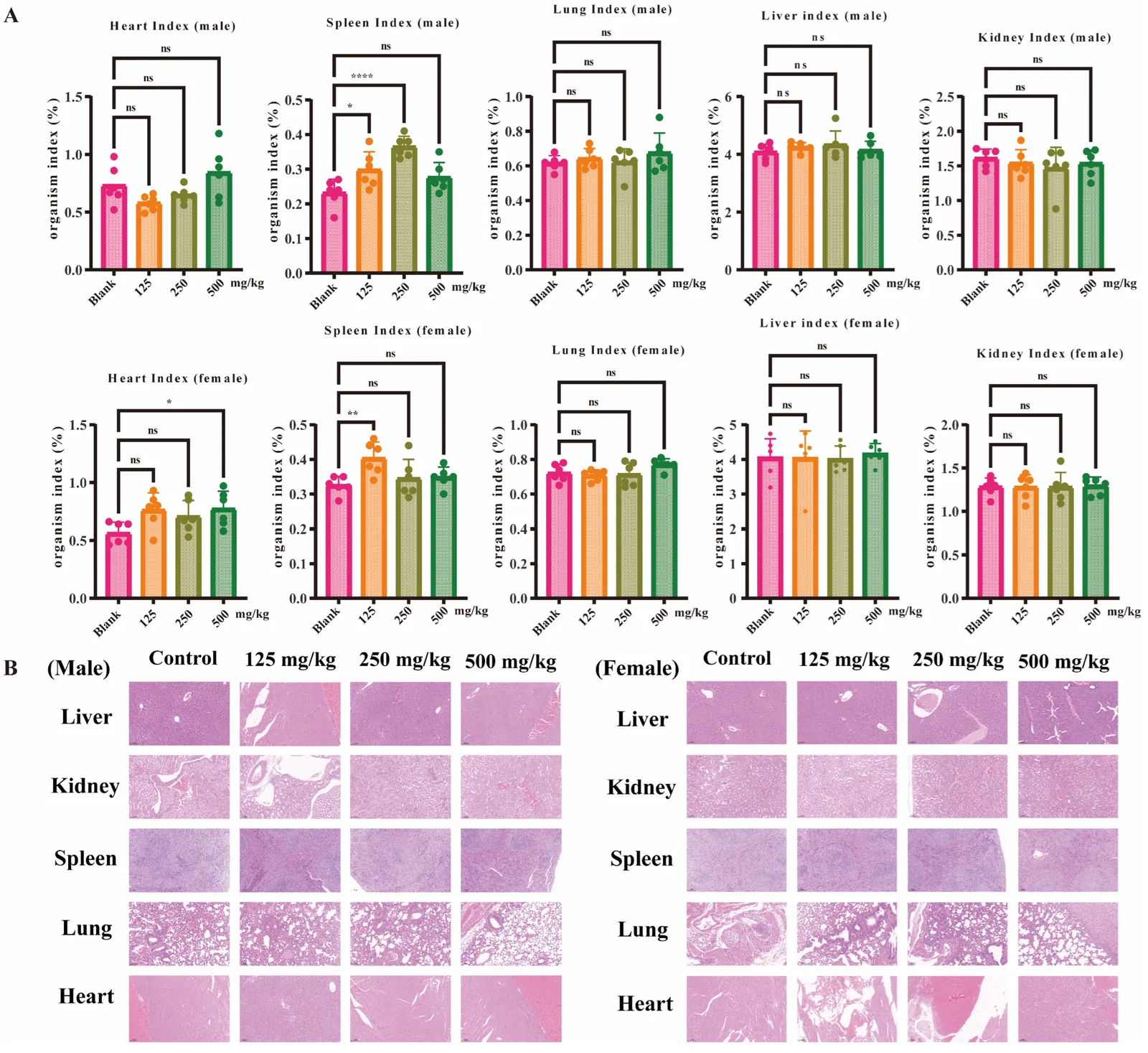
(**A**) Relative organ indices (liver, kidney, spleen, lung, and heart) of mice evaluated at necropsy. (**B**) Representative H&E-stained histopathological matrix of core organs from the control and TPCOM-treated (125, 250, and 500 mg/kg) groups. Data are presented as mean ± SEM. * *p* < 0.05, ** *p* < 0.01, **** *p* < 0.0001; ns, not significant.

Organ indices (Figure 4A) and histopathological examination (Figure 4B) further revealed tissue alterations associated with repeated TPCOM exposure. The spleen index of both male and female mice was significantly increased at 125 and 250 mg/kg/day (*p* < 0.05 to *p* < 0.0001), suggesting alterations in spleen-related physiological status. Histological examination revealed hepatic cytoplasmic vacuolization and microvascular lipid droplet accumulation in mice receiving 500 mg/kg/day. Renal alterations, including degeneration and shedding of renal tubular epithelial cells, were also observed. In addition, alveolar septal thickening and inflammatory cell infiltration were detected in the lungs, while focal disarrangement of myocardial fibers was observed in the heart. Overall, repeated TPCOM exposure for 28 days was associated with biochemical changes and qualitative histopathological alterations involving multiple organs, accompanied by changes in coagulation parameters and glucose- and lipid-metabolism-related indicators.

### 3.3. Network Toxicology Analysis of Potential Molecular Targets and Pathways Associated with TPCOM-Related Organ Toxicity

#### 3.3.1. Potential Molecular Targets and Pathways Associated with TPCOM-Related Hepatic Responses

**Figure 5:**
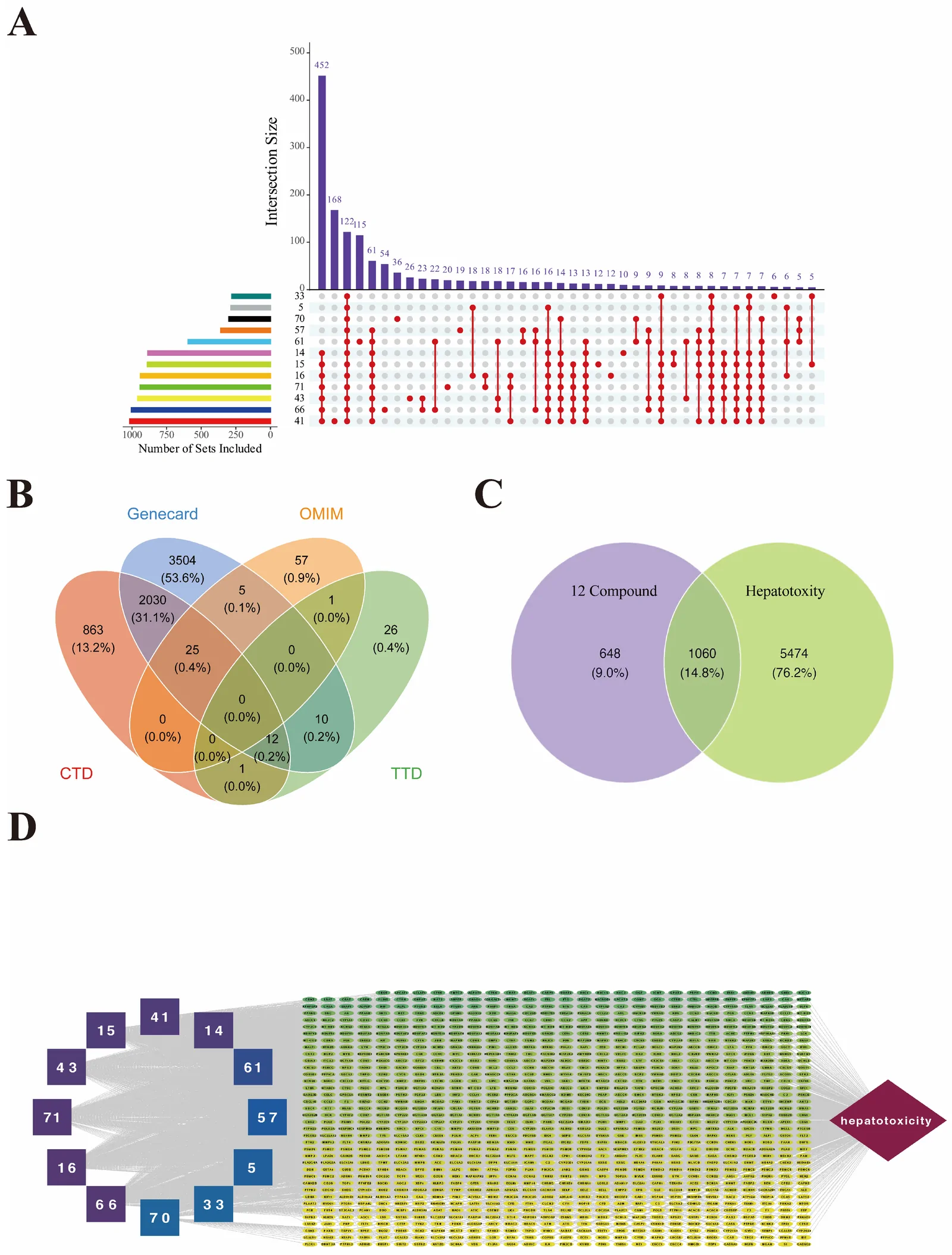
Network toxicology analysis of potential molecular targets and pathways associated with TPCOM-related hepatic responses. (**A**) UpSet plot showing the distribution of the 1708 targets associated with the 12 potential hepatotoxic components. (**B**) Venn diagram of hepatotoxicity-related disease targets retrieved from the GeneCards, OMIM, CTD, and TTD databases. (**C**) Venn diagram intersecting compound-related targets and disease targets to identify 1060 candidate targets for hepatotoxicity. (**D**) The “component-target-disease” interaction network. In panel B, different colors distinguish the four database-derived target sets. In panel C, purple and green represent compound-related targets and hepatotoxicity-related targets, respectively, and the overlapping region represents shared targets.

**Figure 6:**
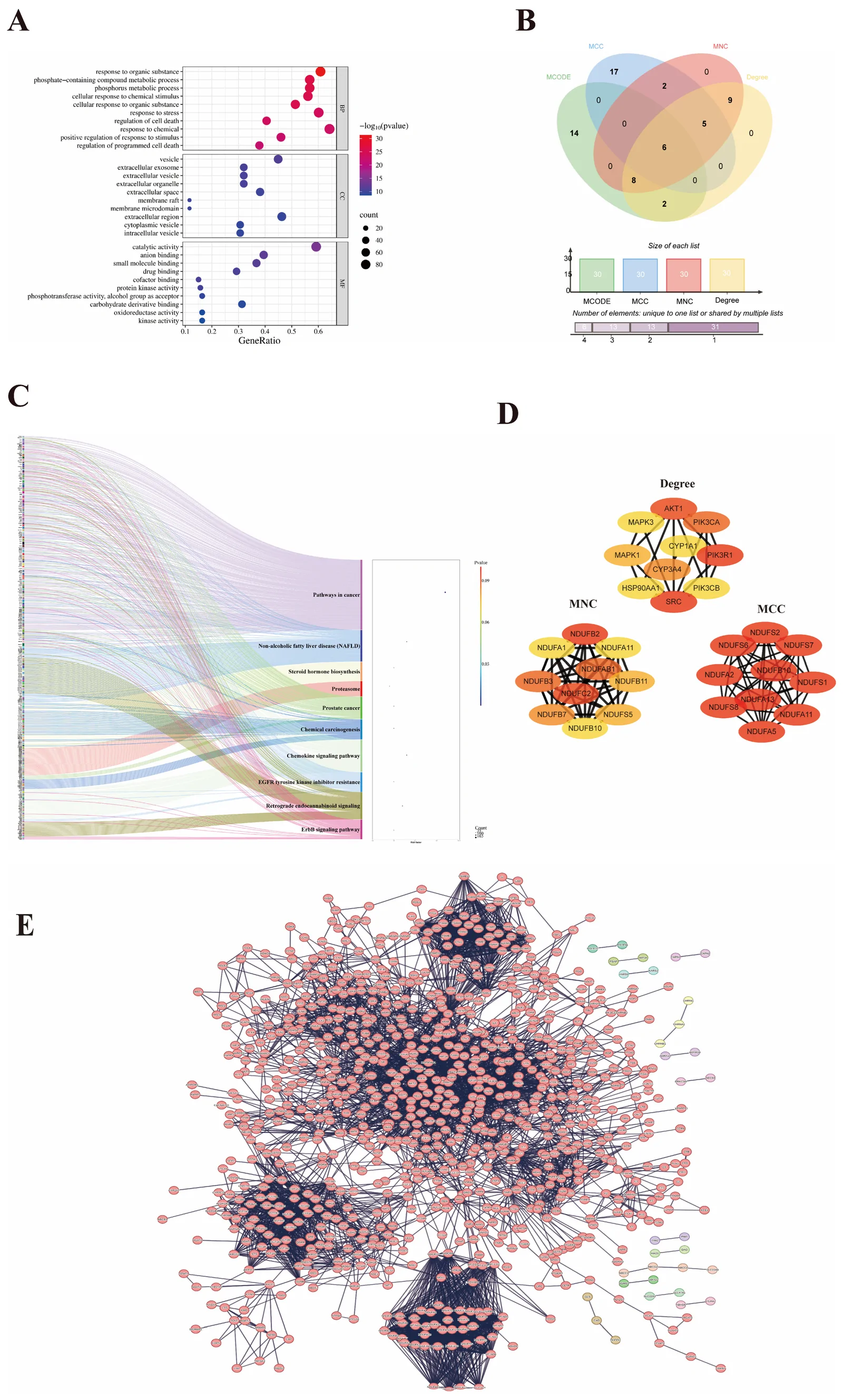
(**A**) Gene Ontology (GO) functional enrichment analysis of the candidate targets, covering biological processes, cellular components, and molecular functions. (**B**) Venn diagram illustrating the intersection of six core hub targets (AKT1, CYP2E1, SRC, NDUFS7, NDUFS3, and NDUFV2) screened via topological algorithms (MCC, MNC, and Degree) in combination with the MCODE clustering algorithm. (**C**) Sankey diagram illustrating the KEGG pathway enrichment analysis. (**D**) Subnetworks of the PPI highlighting the key modules extracted by specific topological algorithms (Degree, MNC, and MCC). (**E**) Protein–protein interaction (PPI) network of the overlapping targets constructed via the STRING database.

To explore the potential molecular mechanisms associated with the hepatotoxic effects of TPCOM, 1708 component-related targets were identified for the 12 potential hepatotoxic components through multi-database integration (Figure 5A). Intersection with 6534 hepatotoxicity-related disease targets (Figure 5B) yielded 1060 overlapping targets potentially associated with TPCOM-related hepatotoxic responses (Figure 5C). Based on these overlapping targets, a component-target-disease interaction network was constructed (Figure 5D). Gene Ontology (GO) enrichment analysis (Figure 6A) showed that the candidate targets were significantly enriched in biological processes related to cellular responses to stress and regulation of programmed cell death. At the molecular function level, protein kinase activity and catalytic activity were among the major enriched categories, indicating that signaling regulation and enzymatic processes may be involved in the biological responses associated with TPCOM exposure.

KEGG pathway enrichment analysis (Figure 6C) further identified several pathways potentially relevant to hepatic responses, including pathways related to non-alcoholic fatty liver disease (NAFLD), chemical carcinogenesis, and ErbB signaling. Notably, multiple genes associated with mitochondrial oxidative phosphorylation, including members of the NDUFS family, as well as the phase I metabolic enzyme CYP2E1, were enriched in the NAFLD-related pathway. These findings suggest that mitochondrial energy metabolism, xenobiotic metabolism, and lipid-associated processes may represent potential biological processes involved in TPCOM-associated hepatic responses. However, these pathway enrichment results primarily indicate potential functional associations and do not by themselves establish a causal relationship between these pathways and TPCOM-induced hepatotoxicity.

A protein–protein interaction (PPI) network was subsequently constructed using the STRING database (Figure 6E). Three topological algorithms, including MCC, MNC, and Degree, were applied to identify highly connected nodes and key subnetworks within the PPI network (Figure 6D), followed by cross-screening with the MCODE clustering algorithm (Figure 6B). Six hub targets were consistently identified: AKT1, CYP2E1, SRC, NDUFS7, NDUFS3, and NDUFV2. These hub targets included both signaling-related proteins and components of the mitochondrial respiratory chain, suggesting potential interactions between signal transduction, xenobiotic metabolism, and mitochondrial energy metabolism in the biological responses to TPCOM exposure. Collectively, the network toxicology analysis identified several candidate molecular targets and pathways that may contribute to TPCOM-associated hepatic alterations, providing a mechanistic framework for subsequent molecular docking and metabolomics analyses.

#### 3.3.2. Potential Molecular Targets and Pathways Associated with TPCOM-Related Renal Responses

**Figure 7:**
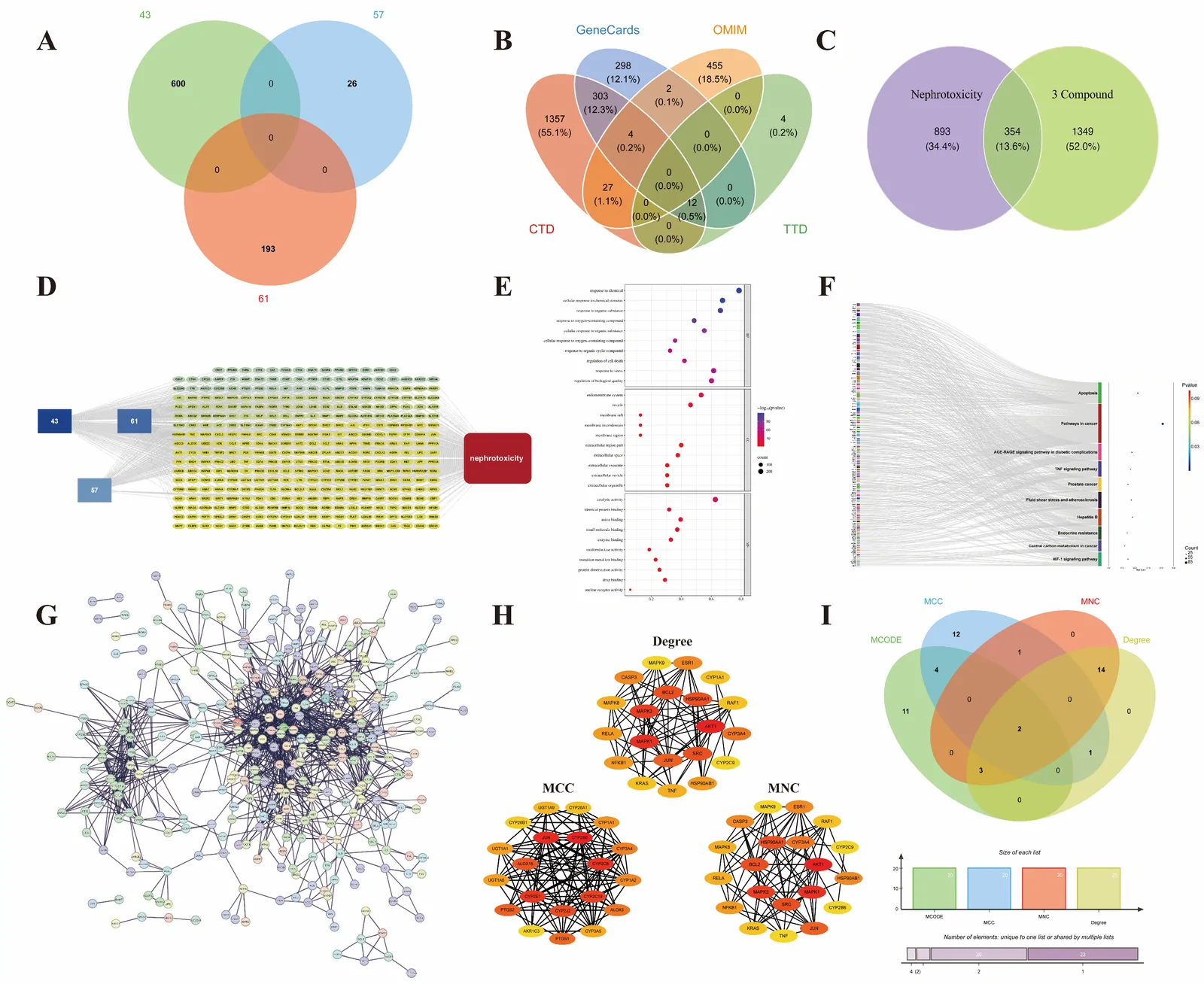
Network toxicology analysis of potential molecular targets and pathways associated with TPCOM-related renal responses. (**A**) Venn diagram showing the distribution of targets for the three key nephrotoxic components. (**B**) Venn diagram of nephrotoxicity-related disease targets retrieved from the GeneCards, OMIM, CTD, and TTD databases. (**C**) Venn diagram intersecting component-related targets and disease targets to identify 354 candidate targets for nephrotoxicity. (**D**) The component-target-disease interaction network. (**E**) Gene Ontology (GO) functional enrichment analysis of the candidate targets. (**F**) Sankey diagram illustrating the KEGG pathway enrichment analysis. (**G**) Protein–protein interaction (PPI) network of the overlapping targets. (**H**) Subnetworks of the PPI highlighting the key modules extracted by specific topological algorithms (Degree, MCC, and MNC). (**I**) Venn diagram illustrating the intersection of five core hub targets (JUN, MAPK3, CYP3A4, RELA, and MAPK1) screened via topological algorithms (MCC, MNC, and Degree) in combination with the MCODE clustering algorithm. In panel B, different colors distinguish the target sets retrieved from the GeneCards, OMIM, CTD, and TTD databases. In panel C, purple and green represent nephrotoxicity-related targets and component-related targets, respectively, with the overlapping region indicating shared targets. In panel I, different colors distinguish the hub-target sets identified using the indicated topological algorithms, and the overlapping regions represent shared targets.

After characterizing the potential mechanisms associated with TPCOM-induced hepatotoxicity, a network toxicology analysis was further performed to explore the molecular basis of nephrotoxicity, another major target organ involved in xenobiotic elimination. Compared with the hepatotoxicity-associated network, the nephrotoxicity-related network showed a greater enrichment of targets involved in inflammatory regulation and apoptosis-related processes. Network-based analysis of the three key nephrotoxic components identified 1349 component-related targets (Figure 7A) and 893 nephrotoxicity-associated disease targets (Figure 7B), resulting in 354 overlapping candidate targets (Figure 7C). Based on these targets, a component-target-disease interaction network was constructed (Figure 7D).

GO enrichment analysis (Figure 7E) indicated that nephrotoxicity-associated targets were mainly involved in cellular stress responses and regulation of programmed cell death, while molecular function analysis showed enrichment in kinase-related activities. KEGG pathway analysis (Figure 7F) further revealed significant enrichment of pathways associated with apoptosis, TNF signaling, AGE-RAGE signaling, and HIF-1 signaling. The enrichment of key genes, including TNF, CASP3, and MAPK8, suggested that inflammatory regulation, oxidative stress-related responses, and apoptosis-associated processes may contribute to TPCOM-associated renal toxicity.

A protein–protein interaction (PPI) network was subsequently constructed using the overlapping targets (Figure 7G). Based on multiple topological algorithms (Degree, MCC, and MNC; Figure 7H) combined with MCODE clustering analysis (Figure 7I), five hub targets were identified: JUN, MAPK3, CYP3A4, RELA, and MAPK1. These targets are mainly associated with transcriptional regulation and MAPK-related signaling processes. Collectively, these network-based predictions suggest that TPCOM-associated nephrotoxicity may involve inflammatory regulation, stress response pathways, and apoptosis-related mechanisms. These findings provide potential molecular clues for interpreting renal alterations observed in vivo, although further experimental validation is required to establish causal relationships.

### 3.4. Evaluation of Component-Target Interactions Based on Molecular Docking

To further investigate the potential interactions between selected TPCOM components and the hub targets identified by network toxicology, molecular docking simulations were performed using AutoDock Vina. Docking scores were used to compare the predicted favorability of ligand–target interactions, with more negative values indicating more favorable predicted interactions. Based on the criteria adopted in this study, docking scores of ≤−4.25 kcal/mol, ≤−5.00 kcal/mol, and ≤−7.00 kcal/mol were considered indicative of potential binding activity, favorable predicted binding, and particularly strong predicted interactions, respectively. These criteria were used for descriptive comparison of the computational docking results and should not be interpreted as experimental measurements of binding affinity.

**Figure 8:**
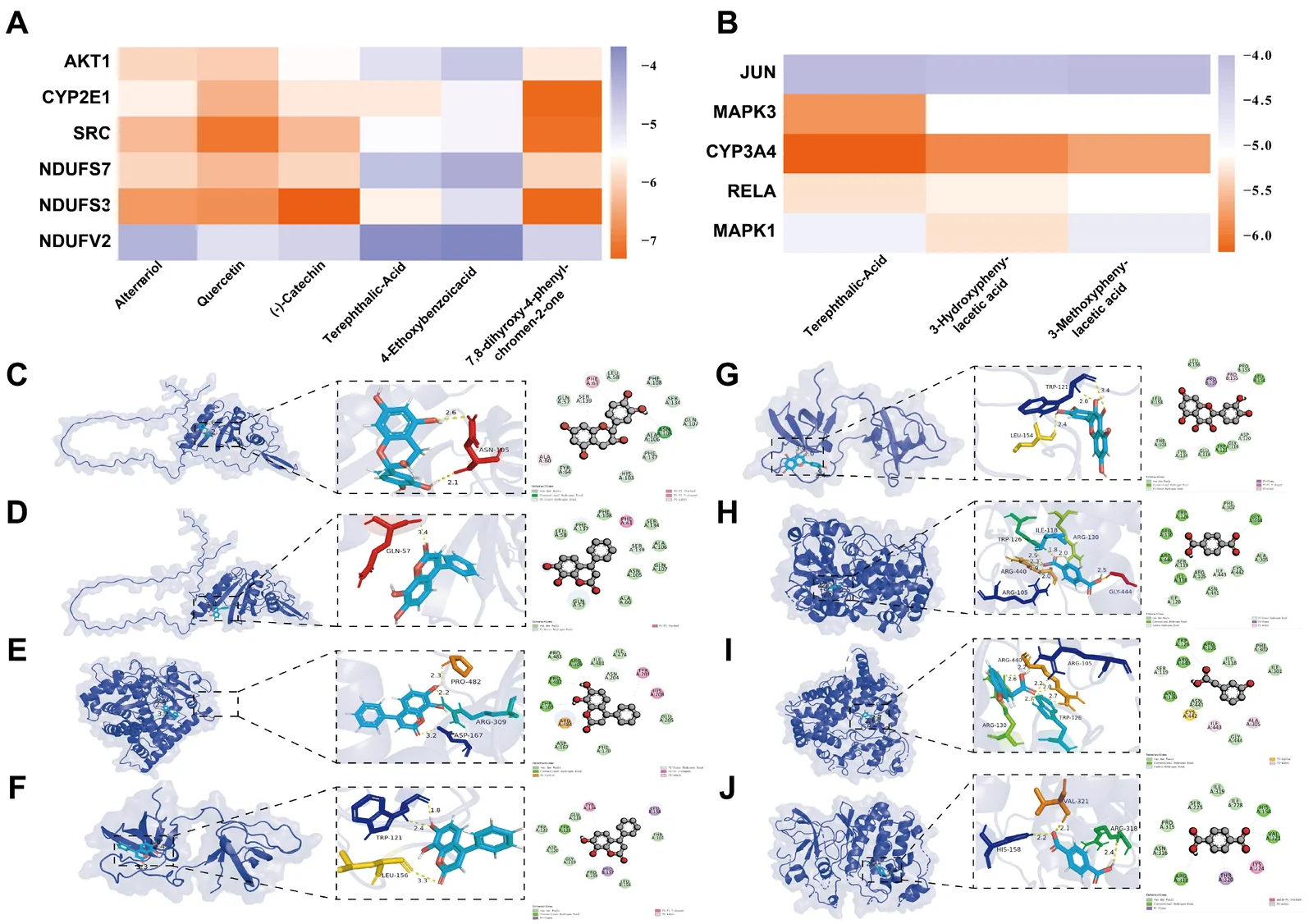
Molecular docking analysis of selected TPCOM components with hepatotoxicity- and nephrotoxicity-related hub targets. (**A**,**B**) Heatmaps showing the AutoDock Vina docking scores (kcal/mol) between selected TPCOM components and (**A**) hepatotoxicity-related hub targets (AKT1, CYP2E1, SRC, NDUFS7, NDUFS3, and NDUFV2) and (**B**) nephrotoxicity-related hub targets (JUN, MAPK3, CYP3A4, RELA, and MAPK1). More negative docking scores indicate more favorable predicted ligand–target interactions. Based on the criteria used in this study, docking scores of ≤−5.0 kcal/mol indicate favorable predicted interactions, whereas scores of ≤−7.0 kcal/mol indicate particularly strong predicted interactions. (**C**–**G**) Representative three-dimensional (3D) and two-dimensional (2D) docking conformations of five hepatotoxicity-related ligand–target complexes with the lowest docking scores. (**H**–**J**) Representative 3D and 2D docking conformations of three nephrotoxicity-related ligand–target complexes. The docking models illustrate the predicted binding poses and key amino acid residues involved in ligand–target interactions, including hydrogen bonds and hydrophobic interactions. These docking results represent computational predictions and do not constitute experimental evidence of direct target binding or functional modulation. In panels (**A**,**B**), heatmap colors represent docking scores according to the corresponding color scales. In panels (**C**–**J**), the colors distinguish the molecular structures and interaction features shown in the docking visualizations.

For the hepatotoxicity-related targets, docking analysis was performed between the selected compounds and six hub targets, including AKT1, CYP2E1, SRC, NDUFS7, NDUFS3, and NDUFV2 (Figure 8A). Among the evaluated ligand–target pairs, (-)-catechin–NDUFS3 exhibited the lowest docking score (−7.4 kcal/mol). In addition, 7,8-dihydroxy-4-phenyl-chromen-2-one showed particularly favorable predicted interactions with CYP2E1 and NDUFS3 (both −7.3 kcal/mol) and SRC (−7.2 kcal/mol). The quercetin–SRC complex also showed a particularly favorable predicted interaction, with a docking score of −7.1 kcal/mol. These ligand–target pairs met the ≤−7.0 kcal/mol criterion used in this study to describe particularly strong predicted docking interactions. Representative docking conformations are shown in Figure 8C–G. The complete docking scores for the hepatotoxicity-related targets are provided in Supplementary Table S3.

For the nephrotoxicity-related targets, molecular docking analysis was performed between selected compounds and the hub targets JUN, MAPK3, CYP3A4, RELA, and MAPK1 (Figure 8B). Terephthalic acid and 3-hydroxyphenylacetic acid showed predicted interactions with CYP3A4, with docking scores of −6.2 and −5.9 kcal/mol, respectively. In addition, 3-methoxyphenylacetic acid showed a predicted interaction with RELA, with a docking score of −5.1 kcal/mol. Although these interactions did not reach the ≤−7.0 kcal/mol criterion used to describe particularly strong predicted docking interactions, their docking scores indicated favorable predicted interactions under the criteria applied in this study. Representative docking conformations are shown in Figure 8H–J. The complete docking scores for the nephrotoxicity-related targets are provided in Supplementary Table S4.

Overall, the molecular docking analysis identified several energetically favorable predicted interactions between selected TPCOM components and the hub targets identified by network toxicology. In particular, the (-)-catechin–NDUFS3 complex exhibited the lowest docking score among the hepatotoxicity-related ligand–target pairs evaluated. These computational results support the plausibility of interactions between selected TPCOM constituents and the predicted hub targets; however, they do not constitute experimental evidence of direct target binding, enzyme inhibition, target activation, or causal involvement in the toxicological effects observed in vivo.

### 3.5. Molecular Dynamics Simulations

**Figure 9:**
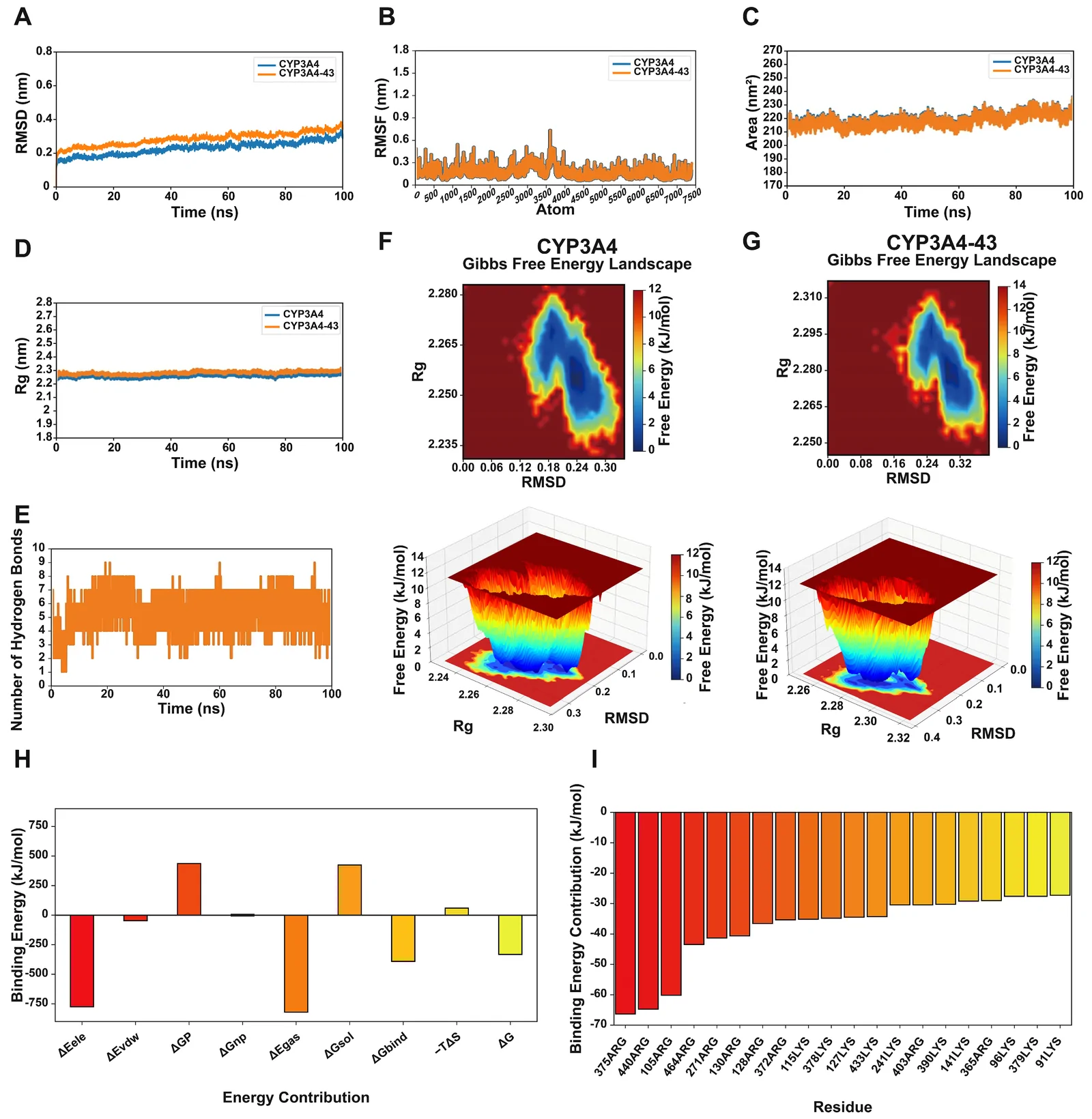
Molecular dynamics analysis of the CYP3A4–terephthalic acid (compound **43**) complex during the 100-ns simulation. (**A**) Time-dependent root-mean-square deviation (RMSD) trajectories of the protein backbone for apo CYP3A4 and the CYP3A4–terephthalic acid complex. (**B**) Root-mean-square fluctuation (RMSF) of amino acid residues during the simulation. (**C**) Solvent-accessible surface area (SASA) trajectories. (**D**) Radius of gyration (Rg) trajectories. (**E**) Time-dependent number of intermolecular hydrogen bonds between CYP3A4 and terephthalic acid. (**F**,**G**) Free-energy landscapes of (**F**) apo CYP3A4 and (**G**) the CYP3A4–terephthalic acid complex constructed using RMSD and Rg as reaction coordinates. (**H**) Estimated binding free energy and individual energetic contributions calculated using the MM/PBSA method. (**I**) Per-residue energy decomposition showing major amino acid residues contributing to the calculated ligand–receptor interaction.

To further evaluate the dynamic stability of the representative ligand–target interactions identified through molecular docking and network toxicology analyses, eight representative complexes were subjected to 100-ns MD simulations. These complexes included ligand–target pairs representing both hepatotoxicity- and nephrotoxicity-related networks. Notably, the (-)-catechin–NDUFS3 complex, which showed the strongest predicted docking interaction in the hepatotoxicity dataset (−7.4 kcal/mol), was included among the MD systems. The complete MD analyses of the representative complexes are provided in Figure 9 and Supplementary Figures S2–S8.

Among the simulated systems, the CYP3A4–terephthalic acid complex (CYP3A4–compound **43**) exhibited favorable conformational stability and binding free-energy characteristics during the 100-ns simulation and was therefore selected as a representative system for detailed presentation in the main text. Importantly, this complex was not selected because it exhibited the strongest AutoDock Vina docking score; rather, its detailed presentation was based on the subsequent MD behavior and its relevance to the predicted xenobiotic-metabolism-related toxicological network.

Trajectory analysis of the CYP3A4–terephthalic acid complex was performed in parallel with that of the unbound CYP3A4 receptor (apo CYP3A4). As shown in Figure 9A, following an initial period of conformational relaxation, the backbone RMSD of the CYP3A4–terephthalic acid complex gradually stabilized and remained at approximately 0.3 nm during the middle and later stages of the simulation. The trajectory showed a generally comparable trend to that of apo CYP3A4, indicating that ligand binding did not induce substantial global structural instability during the simulated period.

The SASA and Rg trajectories (Figure 9C,D) showed relatively limited fluctuations throughout the simulation, suggesting that the overall compactness and solvent exposure of CYP3A4 remained comparatively stable after ligand binding. RMSF analysis (Figure 9B) further showed that most residues surrounding the binding region exhibited relatively limited fluctuations, whereas greater flexibility was mainly observed in terminal and surface loop regions.

Intermolecular hydrogen-bond analysis showed that the ligand and receptor maintained dynamic hydrogen-bond interactions during the simulation (Figure 9E), providing additional support for the persistence of the predicted ligand–receptor interaction. Free-energy landscape analysis (Figure 9F,G) further indicated that the CYP3A4–terephthalic acid complex sampled relatively stable conformational states during the 100-ns trajectory.

MM/PBSA analysis was subsequently used to estimate the binding free energy and its energetic components (Figure 9H). The calculated binding free energy was approximately −350 kJ/mol under the computational conditions employed. Electrostatic and van der Waals interactions contributed favorably to the calculated binding energy, whereas polar solvation opposed complex formation. Per-residue energy decomposition (Figure 9I) further identified several residues, including Arg375 and Arg440, as important contributors to the calculated ligand–receptor interaction.

Collectively, the MD results support the dynamic stability of the predicted CYP3A4–terephthalic acid interaction under the simulation conditions. However, these computational findings should be interpreted as evidence supporting the plausibility of a molecular interaction rather than direct demonstration of CYP3A4 binding, inhibition, or functional modulation in vivo.

### 3.6. Untargeted Metabolomics Identifies Metabolic Alterations Associated with TPCOM-Induced Toxicity

**Figure 10:**
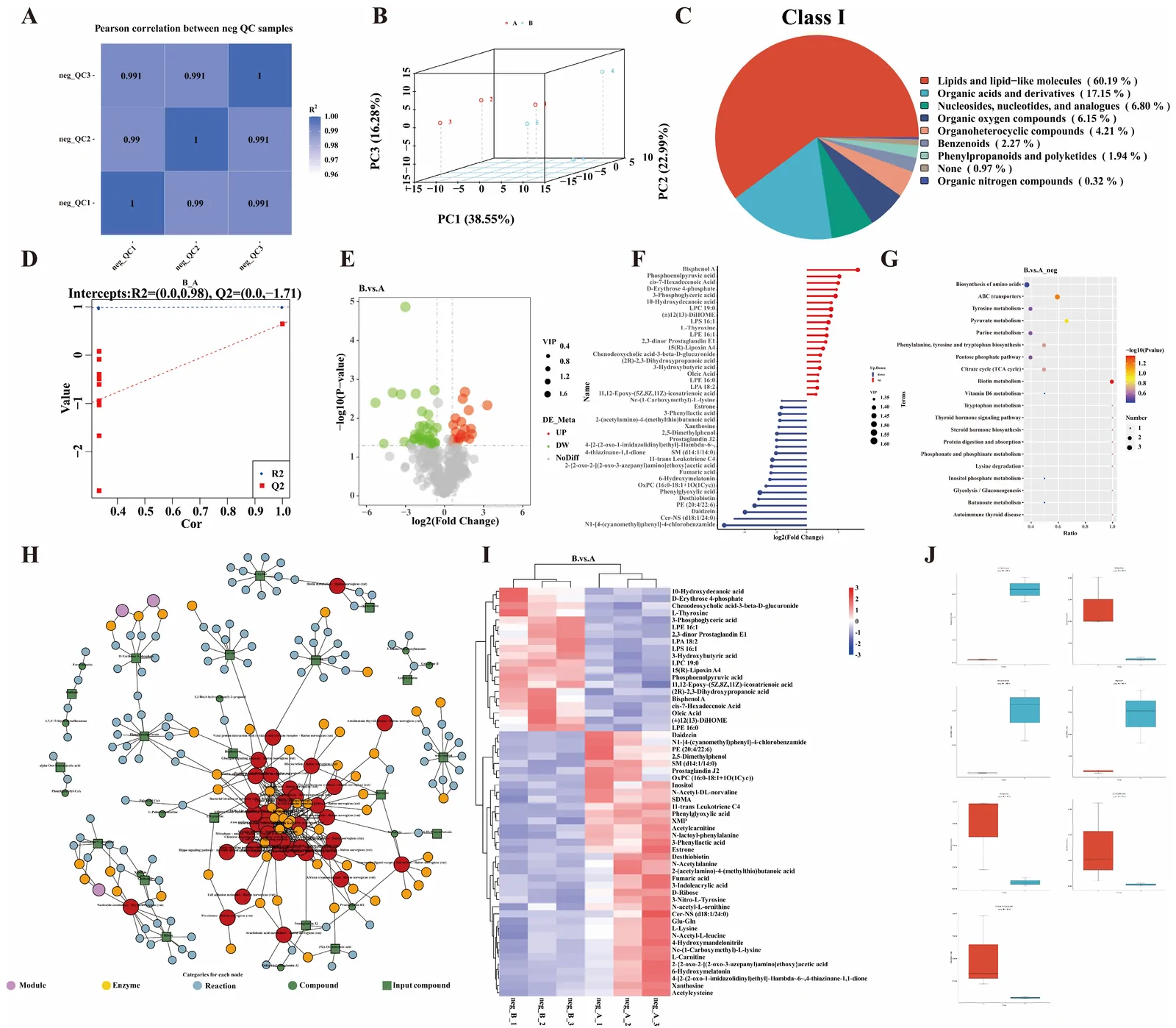
Untargeted metabolomics analysis of early plasma metabolic alterations following a single high-dose TPCOM exposure in male mice. (**A**) Pearson correlation heatmap of the quality control (QC) samples. (**B**) 3D-PCA score plot of the control and TPCOM-treated groups. (**C**) Pie chart showing the chemical ontology classification of the perturbed metabolites. (**D**) Permutation test (*n* = 200) of the PLS-DA model validating its reliability. (**E**) Volcano plot showing exploratory candidate differentially abundant metabolites between the treated and control groups. (**F**) Stem plot highlighting representative candidate differential metabolites based on VIP scores. (**G**) Exploratory KEGG pathway enrichment analysis based on the candidate differential metabolites. (**H**) Metabolite–target/pathway interaction network. (**I**) Hierarchical clustering heatmap showing the abundance patterns of candidate differential metabolites. (**J**) Boxplots displaying the relative abundance of the seven candidate metabolites selected for further evaluation.

To further characterize the systemic metabolic changes associated with TPCOM exposure, untargeted metabolomics analysis was performed using plasma samples collected from C57BL/6 mice. First, Pearson correlation analysis of quality control (QC) samples (Figure 10A) and the three-dimensional principal component analysis (3D-PCA) score plot (Figure 10B) demonstrated tight clustering of QC samples, indicating good analytical reproducibility and stability. The control and TPCOM-treated groups (500 mg/kg) showed distinct separation in the PCA score plot, suggesting substantial alterations in the overall plasma metabolic profiles following high-dose TPCOM exposure. Chemical ontology classification showed that the altered metabolites were mainly categorized as lipids and lipid-like molecules (60.19%) and organic acids and derivatives (17.15%) (Figure 10C), indicating that lipid-related and organic acid-related metabolites represented major categories among the observed metabolic changes.

Partial least squares-discriminant analysis (PLS-DA) was subsequently performed to further evaluate metabolic differences between groups. The 200-iteration permutation test (Figure 10D) generated a Q^2^ regression intercept of −1.71, suggesting acceptable model robustness under the current analytical conditions. Using the exploratory criteria of VIP > 1.0, |FC| ≥ 1.5, and nominal *p* < 0.05, a total of 167 candidate differentially abundant metabolites were identified. After Benjamini–Hochberg false discovery rate (FDR) correction, two metabolites remained significant at q < 0.05: (±)-abscisic acid in positive-ion mode and phenylglyoxylic acid in negative-ion mode. Accordingly, the broader set of 167 metabolites was regarded as exploratory and was retained for subsequent hypothesis-generating pathway and network analyses. Volcano plot analysis (Figure 10E) and stem plot visualization (Figure 10F) highlighted several representative candidate metabolites, including increased levels of abscisic acid (ABA), decanoylcarnitine, and phosphoenolpyruvate (PEP), as well as decreased abundance of sphingolipid-related metabolites such as Cer-NS. Hierarchical clustering analysis (Figure 10I) further demonstrated distinct metabolic patterns between the control and TPCOM-treated groups, suggesting broad metabolic perturbations following TPCOM exposure.

Exploratory KEGG pathway enrichment analysis based on the broader candidate metabolite set revealed associations with pathways related to the tricarboxylic acid (TCA) cycle, pyruvate metabolism, and amino acid metabolism (Figure 10G), suggesting potential alterations in systemic energy metabolism and intermediary metabolic processes. Furthermore, the metabolite–target/pathway network (Figure 10H) suggested possible associations between the candidate metabolites and signaling pathways including PI3K-Akt, AMPK, and Toll-like receptor (TLR) signaling. Combined with the network toxicology analysis, these findings provide hypothesis-generating evidence suggesting that metabolic dysregulation involving energy metabolism, lipid metabolism, and inflammation-related pathways may participate in the biological responses associated with high-dose TPCOM exposure. Nevertheless, these associations require further experimental validation to clarify their biological significance.

Finally, seven metabolites associated with the observed metabolic response were selected as candidate biomarkers for further evaluation. Boxplot analysis (Figure 10J) demonstrated differences in the abundance of these metabolites between the control and TPCOM-treated groups. These metabolites may serve as potential indicators of TPCOM-associated metabolic disturbances; however, their diagnostic performance, specificity, and reproducibility require targeted quantitative validation and confirmation in independent experimental datasets.

## 4. Discussion

*Cydonia oblonga* Mill. fruits have attracted considerable attention as traditional food and medicinal resources because of their rich polyphenolic composition and reported antioxidant, anti-inflammatory, and metabolic activities [32,33]. Previous studies have generally suggested favorable tolerance of some *Cydonia oblonga* preparations under specific experimental conditions. Rahman et al. reported that an aqueous–methanolic seed extract was tolerated in mice at doses up to 4 g/kg and produced no major blood chemistry alterations during sub-chronic exposure [7], while Aslam and Sial reported no apparent acute toxicity of a hydroalcoholic fruit extract at doses up to 3000 mg/kg in rats. Nevertheless, direct comparison with the present findings should be made cautiously because the tested preparations differed in plant part, extraction method, chemical composition, and exposure regimen. The toxicological effects observed here under high-dose TPCOM exposure may therefore reflect the specific composition and enrichment of the polyphenolic fraction rather than the toxicity profile of quince preparations in general. However, the toxicological characteristics of TPCOM under excessive exposure conditions remain insufficiently understood. In the present study, an integrated evaluation framework combining in vivo toxicological assessment, network toxicology, molecular docking and molecular dynamics simulations, and untargeted plasma metabolomics was established to characterize the biological responses associated with high-dose TPCOM exposure. UHPLC-QE-MS analysis enabled the annotation of 468 chemical features, from which 89 selected phenolic and related constituents were selected for subsequent toxicological analysis based on integrated screening criteria [34,35]. Acute toxicity assessment revealed significant toxicity during high-dose exposure, with mortality observed in preliminary evaluations at 5000 and 2500 mg/kg, while 1000 mg/kg was selected for the subsequent acute toxicity study. The 28-day repeated-dose study further demonstrated treatment-associated biochemical and histopathological alterations involving multiple organs, particularly the liver and kidneys, together with disturbances in glucose–lipid metabolism and coagulation-related parameters. Collectively, these findings provide toxicological evidence supporting the safety evaluation of TPCOM and highlight the importance of characterizing polyphenol-rich food-derived ingredients under excessive exposure conditions [36,37,38].

It should be noted that the PPI network was constructed using human protein-interaction data, whereas the in vivo toxicity experiments were conducted in C57BL/6 mice. Accordingly, the computationally identified hub targets and pathways should be interpreted as hypothesis-generating, human-relevant molecular associations rather than direct mechanistic confirmation of the mouse phenotypes. Although many core signaling and metabolic proteins are conserved across species, species-specific differences in gene regulation, protein interactions, and xenobiotic metabolism may affect the correspondence between the human network predictions and the mouse in vivo responses. Future studies incorporating mouse ortholog mapping and direct experimental validation in relevant tissues are warranted.

The use of a consistent C57BL/6 mouse model across the acute toxicity, 28-day repeated-dose toxicity, and untargeted metabolomics experiments enabled integrated evaluation of toxicological and metabolic responses under a comparable genetic background. The inclusion of both male and female mice further facilitated the exploration of potential sex-related variations across multiple endpoints. However, the present study was not specifically designed to investigate sex-by-dose interactions, and therefore the observed differences between sexes should be interpreted as sex-associated responses rather than definitive evidence of differential susceptibility to TPCOM. Nevertheless, the additional two-way ANOVA indicated significant sex × treatment interactions for several endpoints, including APTT, GLU, and TG, supporting sex-related differences in treatment responses for these specific parameters. Moreover, the use of a single mouse strain may restrict the extrapolation of these findings to other genetic backgrounds and species. Future studies incorporating additional animal models and appropriately powered sex-stratified designs are needed to validate whether the observed sex-related responses represent consistent differences in TPCOM toxicity.

Following the identification of the toxicological phenotypes, network toxicology and molecular docking analyses were conducted to explore potential molecular targets and signaling pathways associated with TPCOM exposure. The integrated network analysis revealed several polyphenolic constituents and protein targets that may be involved in the biological responses induced by TPCOM. For instance, quercetin, a flavonoid with well-documented antioxidant and hepatoprotective activities under specific experimental conditions [39], was predicted to interact with several hepatotoxicity-associated targets in the computational analysis [40,41]. In addition, CYP2E1, a key xenobiotic-metabolizing enzyme involved in oxidative stress regulation and lipid peroxidation processes under various toxicological conditions [42,43,44], was identified as a potential target associated with TPCOM-induced hepatic alterations. These findings suggest that modulation of xenobiotic metabolism and oxidative stress-related pathways may represent potential mechanisms underlying TPCOM-associated hepatotoxic responses. However, CYP2E1 enzymatic activity, reactive oxygen species production, and lipid peroxidation levels were not directly measured in the present study; therefore, these predicted associations require further experimental validation.

Similarly, computational predictions suggested that certain TPCOM constituents, including terephthalic acid, may interact with renal toxicity-associated targets, providing potential molecular clues for the observed renal alterations. However, the contribution of individual compounds to renal injury remains unclear and requires targeted validation using isolated compounds or mechanistic experiments. PPI network analysis further identified several hub proteins, including AKT1, CYP2E1, and JUN, which are involved in cellular stress responses, apoptosis regulation, and signal transduction [45,46,47]. These findings provide potential molecular connections between predicted targets and the toxicological phenotypes observed in vivo. Nevertheless, the network toxicology and docking results should be interpreted as hypothesis-generating evidence rather than definitive molecular mechanisms, and the causal involvement of these targets in TPCOM-induced organ injury remains to be experimentally confirmed.

It should be emphasized that the untargeted metabolomics experiment was conducted in male mice following a single oral dose of 500 mg/kg TPCOM, with plasma collected at 60 min post-administration, whereas the principal repeated-dose toxicity study involved daily administration for 28 days in both male and female mice. Accordingly, the metabolomic alterations observed in the present study primarily reflect an early systemic metabolic response to acute high-dose exposure and should not be interpreted as a direct representation of the metabolic consequences of 28-day repeated exposure. In addition, because only male mice were included in the metabolomics experiment, these data cannot be used to infer sex-specific metabolic responses.

The target-based predictions from network toxicology were further integrated with untargeted plasma metabolomics analysis in C57BL/6 mice to explore metabolic alterations associated with TPCOM exposure. The altered plasma metabolic profiles revealed that high-dose TPCOM treatment was associated with substantial systemic metabolic disturbances [48,49]. Several differential metabolites, including abscisic acid, decanoylcarnitine, and phosphoenolpyruvate, were enriched in pathways related to the tricarboxylic acid (TCA) cycle, amino acid metabolism, and energy homeostasis. In addition, metabolite-associated pathway analysis suggested potential involvement of signaling processes related to PI3K-Akt and AMPK pathways. Disturbances in energy metabolism are closely linked to cellular stress responses and metabolic adaptation, which are consistent with the stress-related functions of hub targets such as JUN and AKT1 identified in the network analysis. Previous studies have suggested that oxidative stress, mitochondrial dysfunction, and apoptosis-related signaling may contribute to organ toxicity under certain exposure conditions [50,51]. Therefore, the integrated findings indicate that metabolic imbalance, altered xenobiotic responses, and stress-associated signaling pathways may collectively participate in the toxicological responses observed following high-dose TPCOM exposure. However, the specific molecular mechanisms linking these metabolic alterations to tissue injury require further experimental confirmation.

A further limitation of the present study is that the histopathological assessment was primarily qualitative and was not supported by standardized blinded semiquantitative lesion scoring. Therefore, the histological findings should be interpreted as descriptive evidence of treatment-associated tissue alterations rather than quantitative evidence of lesion severity or dose dependence. Future studies should incorporate standardized blinded lesion-scoring systems to strengthen the quantitative evaluation of tissue injury.

Statistically significant changes in individual biochemical parameters were not automatically interpreted as adverse effects. In particular, reductions in ALT and AST were considered findings of uncertain toxicological significance unless supported by concordant evidence from other endpoints. The toxicological interpretation was therefore based on an integrated evaluation of clinical, biochemical, organ-weight, coagulation, and histopathological findings.

Furthermore, multivariate analysis enabled the identification of a candidate seven-metabolite panel associated with TPCOM exposure. These metabolites may serve as potential indicators for monitoring TPCOM-related metabolic disturbances; however, targeted quantitative validation and evaluation in independent experimental cohorts are required before their utility as reliable biomarkers can be established.

## 5. Conclusions

This study demonstrates that high-dose exposure to TPCOM produces acute and 28-day repeated-dose toxicological effects in C57BL/6 mice, with the liver and kidneys emerging as important target organs. The observed alterations in biochemical parameters, glucose and lipid metabolism, coagulation indices, and histopathology indicate that TPCOM exposure can induce systemic metabolic and tissue disturbances, with sex-dependent differences observed across several toxicological endpoints. Integrated chemical profiling, network toxicology, molecular dynamics simulations, and untargeted plasma metabolomics further identified potential molecular and metabolic pathways associated with TPCOM-induced toxicity. Although these computational and metabolomic findings provide mechanistic insights, the predicted molecular interactions and downstream pathways require further experimental validation. Seven metabolites were identified as candidate biomarkers associated with the toxicological response; however, their diagnostic performance should be confirmed in independent cohorts. The doses used in the present study were selected primarily for toxicological hazard identification under high-dose exposure conditions and were not intended to reproduce typical human dietary exposure. Because standardized intake recommendations and reliable human exposure data for TPCOM preparations are currently limited, and because the composition of *Cydonia oblonga* products may vary among preparations, direct quantitative extrapolation of the mouse doses to human consumption would be inappropriate. Therefore, the toxicological findings observed in this study should not be interpreted as evidence of risk associated with normal dietary intake or functional food use. Further studies using standardized preparations, realistic exposure levels, pharmacokinetic data, and longer-term low-dose administration are needed to support human health risk assessment. Collectively, these findings provide a systematic toxicological basis for evaluating the safety of TPCOM under high-dose exposure conditions and support further investigation of its potential risks and biological effects when used as a functional food or natural product ingredient.

## Data Availability

The original contributions presented in this study are included in the article/Supplementary Materials. Further inquiries can be directed to the corresponding author.

## Supplementary Materials

The following supporting information can be downloaded at: https://www.mdpi.com/article/10.3390/foods15203737/s1, Figure S1: Chemical structures of the 12 TPCOM compounds selected for molecular docking; Figures S2–S8: Additional molecular dynamics simulation results for the remaining selected TPCOM compound–target complexes; Table S1: Chemical identification information for compounds identified in TPCOM by UHPLC-QE-MS; Table S2: Relative abundance and abundance ranking of identified compounds in TPCOM; Table S3: Molecular docking binding energy results for hepatotoxicity-related hub targets; Table S4: Molecular docking binding energy results for nephrotoxicity-related hub targets; Table S5: Pharmacokinetic properties and screening criteria for candidate TPCOM compounds; Table S6: Receptor structure identifiers and AutoDock Vina grid parameters used for molecular docking; Table S7: Compound annotations corresponding to numbered peaks in positive- and negative-ion mode TIC chromatograms.
